# Trends and inequalities in the burden of mortality in Scotland 2000–2015

**DOI:** 10.1371/journal.pone.0196906

**Published:** 2018-08-01

**Authors:** Oscar Mesalles-Naranjo, Ian Grant, Grant M. A. Wyper, Diane Stockton, Richard Dobbie, Mag McFadden, Elaine Tod, Neil Craig, Colin M. Fischbacher, Gerry McCartney

**Affiliations:** 1 Public Health Intelligence, NHS National Services Scotland, Gyle Square, Edinburgh, Scotland; 2 NHS Health Scotland, Gyle Square, Edinburgh, Scotland; Sciensano, BELGIUM

## Abstract

**Background:**

Cause-specific mortality trends are routinely reported for Scotland. However, ill-defined deaths are not routinely redistributed to more precise and internationally comparable categories nor is the mortality reported in terms of years of life lost to facilitate the calculation of the burden of disease. This study describes trends in Years of Life Lost (YLL) for specific causes of death in Scotland from 2000 to 2015.

**Methods:**

We obtained records of all deaths in Scotland by age, sex, area and underlying cause of death between 2000 and 2015. We redistributed Ill-Defined Deaths (IDDs) to more exact and meaningful causes using internationally accepted methods. Years of Life Lost (YLL) using remaining life expectancy by sex and single year of age from the 2013 Scottish life table were calculated for each death. These data were then used to calculate the crude and age-standardised trends in YLL by age, sex, cause, health board area, and area deprivation decile.

**Results:**

Between 2000 and 2015, the annual percentage of deaths that were ill-defined varied between 10% and 12%. The proportion of deaths that were IDDs increased over time and were more common: in women; amongst those aged 1–4 years, 25–34 years and >80 years; in more deprived areas; and in the island health boards. The total YLL fell from around 17,800 years per 100,000 population in 2000 to around 13,500 years by 2015. The largest individual contributors to YLL were Ischaemic Heart Disease (IHD), respiratory cancers, Chronic Obstructive Pulmonary Disease (COPD), cerebrovascular disease and Alzheimer’s/dementia. The proportion of total YLL due to IHD and stroke declined over time, but increased for Alzheimer’s/dementia and drug use disorders. There were marked absolute inequalities in YLL by area deprivation, with a mean Slope Index of Inequality (SII) for all causes of 15,344 YLL between 2001 and 2015, with IHD and COPD the greatest contributors. The Relative Index of Inequality (RII) for YLL was highest for self-harm and lower respiratory infections.

**Conclusion:**

The total YLL per 100,000 population in Scotland has declined over time. The YLL in Scotland is predominantly due to a wide range of chronic diseases, substance misuse, self-harm and increasingly Alzheimer’s disease and dementia. Inequalities in YLL, in both relative and absolute terms, are stark.

## Background

Burden of disease studies aim to quantify the total health deficits arising from premature death and time spent in sub-optimal health states due to illness and injury for specified populations. They are valuable because they can help to identify the disease processes which account for the largest burden, and can also be the basis for identifying the proportion of the burden that can be attributed to a range of exposures. The extent of lost Disability-Adjusted Life Years (DALYs) is the most common metric for comparing the burden of disease, and a key component of this is the calculation of the loss of years of life arising from premature death (Years of Life Lost (YLL)) [1]. Most high-income nations have robust death registration systems which ensure that deaths are routinely recorded, the causes are medically certified and the age at death is accurate. However, even within high income countries the causes of death recorded can be of mixed quality and the recording of ill-defined death (IDD) causes remains widespread and to some extent unavoidable, in that it is not always appropriate to undertake extensive investigation to establish an exact cause of death [2].

The lack of robust comparable data across nations has led to substantial methodological innovation to produce estimates of YLL, YLD and DALYs for all countries over time [3–7]. Some of these approaches create modelled estimates for these parameters, using available data on population demography, mortality, and morbidity in combination with data on the economic and social development of a country (e.g. Gross Domestic Product) [7,8].

This paper reports on the estimation of YLL for the population of Scotland between 2000 and 2015 overall, by sex, cause of death, health board area, and area deprivation. These data will be used in combination with YLD data in due course to calculate an overall burden of disease for Scotland (see http://www.scotpho.org.uk/comparative-health/burden-of-disease/overview/) and to calculate the extent to which burden can be attributed to a range of exposures.

## Methods

We report our results in line with the RECORD statement [9].

### Data inputs

We obtained data on all deaths within Scotland by age, sex, cause of death and postcode from the National Records for Scotland (NRS) for each year from 2000 to 2015. The causes of death were coded using the World Health Organisation (WHO) International Statistical Classification of Diseases and Related Health Problems 10^th^ revision (ICD-10). We could not map deaths coded using ICD-9 (1981–1996) to the available redistribution algorithms and were therefore limited to this later time period for the analysis. For the broader calculation of DALYs, of which the YLL calculations in this paper is part, we were restricted to the period 2000 onwards because we required a 20 year ‘look-back’ period for particular hospitalisation records. We therefore restricted our analysis here to the period from 2000–2015. For the deprivation analysis we restricted the years to 2001–2015. We used the underlying cause from death certificates as selected by NRS using WHO mortality coding rules.

Datazones in Scotland (similarly sized areas of approximately 700 people) each have an allocated deprivation score, the Scottish Index of Multiple Deprivation (SIMD), which is derived from administrative data. Further details of the SIMD methods and data are available at: http://www.gov.scot/Topics/Statistics/SIMD. We used the overall SIMD (i.e. the index using data weighted across all aspects of deprivation included) as this was available across all the relevant datasets, but it incurs a theoretical risk of reverse causality because health outcomes are included as one of the deprivation domains. The geographical boundaries of the datazones were revised in 2001 using the 2001 census data. We therefore restricted our deprivation analysis to 2001–2015 to avoid a discontinuity in the data series. There is however a high correlation between the overall index and the non-health deprivation domains. The SIMD is updated regularly and so we used the most relevant index for each death (SIMD2004 for deaths in 2001–2003; SIMD2006 for 2004–2006; SIMD2009 for 2007–2009; SIMD2012 for 2010–2013; and SIMD2016 for 2014–2015). These datazones are ranked across Scotland and divided into deciles with approximately equal population sizes. Each death was thereby categorised by deprivation based on the place of residence at the time of death. The geographical information contained within the deaths data also allowed us to provide the results for each health board area (the 14 differently-sized administrative units responsible for providing health services) across Scotland.

### Redistribution of IDDs

In common with other burden of disease studies [7,8], we reclassified ill-defined deaths (IDD) to other more precise and meaningful causes. Although others have used the term ‘garbage codes’, we prefer to describe these deaths as ‘ill-defined’, as ‘garbage codes’ implies that the deaths are incorrectly coded and are discarded. We used the redistribution algorithm shared with us by the Institute for Health Metrics and Evaluation (IHME), and adapted it to Scottish data. This algorithm defines a set of IDD types, each of which is redistributed to a number of meaningful causes using either fixed coefficients for the whole population or with different redistributions by age and/or sex strata. The codes which we redistributed from and to are given in Table A in S1 File and the full redistribution algorithm in Table A in S2 File.

### Analysis

YLL were calculated using the remaining life expectancy for a given sex and single year of age from the 2013 Scottish life table as published by the Office for National Statistics (ONS) as the notional maximum life expectancy (of 100 years) [10]. The choice of life table to use as the ‘ideal’ is somewhat arbitrary. We chose to use Scottish life tables rather than IHME or WHO tables because the primary focus of this work was to inform Scottish policymakers with more achievable mortality parameters in the short-run, rather than to make international comparisons. The Scottish life tables also facilitated the use of sex-specific life tables. We used 2013 as the reference year for the whole period, instead of a different life table for each year, to capture in our study the increases in life expectancy over time and the real secular trends in YLL due to specific causes. The alternative to this, using the life table for each specific year would mean that we could not present meaningful trends in YLL for all-causes or specific causes of death. To calculate rates the mid-year population estimates for each year published by National Records of Scotland (NRS) were used as denominators. Where the data were age-standardised, this was done directly using the 2013 European Standard Population.

The Slope Index of Inequality (SII) was calculated as the slope coefficient from a weighted least squares linear regression of the YLL outcome of interest for deprivation deciles across the population. This is interpreted as the difference in YLL between the most and least deprived in the population, accounting for the distribution across the whole population. The Relative Index of Inequality (RII) was calculated as the SII divided by the mean YLL for the population [11], and can be interpreted as the difference between the YLL in the most and least deprived in the population after accounting for the average YLL in the population.

## Results

### Deaths and YLL

The mean annual YLL (for the period 2000–2015) was 726,278 for the total population. The causes which accounted for the 10 greatest mean annual YLL were in order: ischaemic heart disease (113,060 YLL), trachea, bronchus, and lung cancers (60,853 YLL), cerebrovascular disease (51,403 YLL), chronic obstructive pulmonary disease (37,698 YLL), chronic liver diseases including cirrhosis (29,492 YLL), suicide and self-harm related injuries (25,508 YLL), colorectal cancer (24,115 YLL), lower respiratory infections (23,724 YLL), Alzheimer's disease and other dementias (22,037 YLL), and drug use disorders (20,198 YLL).

### Ill-Defined Deaths (IDD) and redistribution

The percentage of deaths that were ill-defined varied between 10 and 12% between 2000 and 2015, with a slight increase since 2000 (Table A in S3 File). IDDs were more common amongst deaths in women than men, although there has been a noticeable increase between 2000 and 2015 in the proportion of deaths that were ill-defined for men (form 9.1% to 11.4%), and by 2015 the proportion of IDD was the same for both men and women (Table B in S3 File). The proportion of deaths which were ill-defined varied markedly by age. IDDs were most common amongst those dying aged 1–4 years and 25–34 years, but were also high over age 80 years (Table C in S3 File). Those aged >75 years accounted for 65% of all IDDs in absolute terms because of the greater number of deaths in that group (Table C in S3 File). The percentage of deaths that were ill-defined increased between 2010 and 2015 from 17% to over 30% for those aged 15–34 years (Table D in S3 File). This increase was slightly higher for males (from 13.2% to 33.5%) than for females (from 13.5% to 29.1%) (Table E in S3 File). The mean percentage of deaths that were ill-defined across NHS Health Boards between 2000 and 2015 ranged from 9% to 14%, with the three island boards (Shetland, Orkney and Western Isles) having the highest percentage of IDDs and NHS Forth Valley and NHS Borders having the lowest percentages (Table F in S3 File). There was only a very slight gradient in IDDs by area deprivation, with the percentage 1.3% higher for men and 0.2% higher for women in the most deprived areas in Scotland compared to the least deprived (Table G in S3 File). The difference between the proportion of IDDs in the least and most deprived areas for men increased suddenly from year 2011 and continued to 2015 (Table H in S3 File), but this is an artefact of a change in how drug-related deaths were coded between 2010 and 2011. Further detail on the change in coding and its impact is provided here: https://www.nrscotland.gov.uk/statistics-and-data/statistics/statistics-by-theme/vital-events/deaths/suicides/the-definition-of-the-statistics.

IDD are deaths whereby the causes recorded by physicians on death certificates do not represent precise underlying causes of death. For the purpose of redistributing IDD, we created IDD types, which group multiple ICD-10 codes (Table A in S1 File). The specific IDD types that accounted for most of the IDDs were:

- Malignant neoplasm of other and ill-defined sites;
- Heart failure and other ill defined cardiovascular conditions, (including cardiomegaly, other pulmonary heart diseases, disease of pulmonary vessels, unspecified, systolic (congestive) heart failure; and Disseminated Intravascular Coagulation;
- Other specified respiratory disorders;
- Streptococcal, severe and other sepsis related infections; (including gas gangrene and gangrene not elsewhere classified; Toxic shock syndrome; Staphylococcal infection, unspecified site);
- Pneumonitis due to solids and liquids;
- Other and unspecified diseases; including unspecified bacterial and infectious diseases, endocrine, nutritional and metabolic diseases; and Mental and behavioural disorders.

These six IDD types accounted for more than 50% of all IDDs between 2000 and 2015 (Table A in S4 File). Between 2000 and 2015 some IDD codes became less common (e.g. ‘Heart failure’, ‘cardiomegaly’, ‘other pulmonary heart diseases’, other diseases of pulmonary vessels’, unspecified, systolic (congestive) heart failure; ‘disseminated intravascular coagulation’ and ‘malignant neoplasm of other and ill- defined sites’) whilst others became more common (e.g. ‘Pneumonitis due to solids and liquids’; ‘accidental poisoning by and exposure to narcotics and psychodysleptics [hallucinogens]’; ‘streptococcal, severe and other sepsis’, ‘gas gangrene and gangrene not elsewhere classified’. Toxic shock’) (Table B in S4 File). Some IDDs were more common amongst men (‘Accidental poisoning by and exposure to narcotics and psychodysleptics [hallucinogens], not elsewhere classified’; ‘Pneumonitis due to solids and liquids’; ‘Malignant neoplasm of other and ill-defined sites’) whilst others were more common amongst women (‘Senility’; ‘Other specified respiratory disorders’ and ‘Heart failure, cardiomegaly, other pulmonary heart diseases, disease of pulmonary vessels, unspecified, systolic (congestive) heart failure; and Disseminated Intravascular Coagulation’) (Table C in S4 File). Amongst the oldest groups, the following IDDs were more common: ‘Malignant neoplasm of other and ill- defined sites’, ‘Other specified respiratory disorders’ and ‘Pneumonitis due to solids and liquids’ were all more common IDDs at older ages. In the first year of life ‘Streptococcal, severe and other sepsis/Gas gangrene and gangrene not elsewhere classified/Toxic shock’; and ‘Other and unspecified bacterial and infectious diseases’, were common IDDs. Finally, ‘Poisoning by and exposure to narcotics and psychodysleptics [hallucinogens], not elsewhere classified’ and ‘Accidental poisoning by and exposure to narcotics and psychodysleptics [hallucinogens], not elsewhere classified’ were more common IDDs amongst those aged 15–49 years (Table D in S4 File). There was also some variation in the use of IDD codes across Scotland (Table E in S4 File) with Orkney, Shetland, Western Isles and Fife Boards having a higher percentage of IDDs for ‘Senility’ than the rest of Boards. Across deprivation deciles ‘Accidental poisoning by and exposure to narcotics and psychodysleptics [hallucinogens], not elsewhere classified’ were more common IDDs in deprived areas whilst ‘Senility’ was more common in least deprived areas–probably reflecting differences in the age profile of the populations (Table F in S4 File).

### Time trends in YLL

We provide both crude and age-standardised YLL data over time (Table A in S5 File). The crude data are often the most appropriate in ascertaining the absolute size of an issue for a population and for service planning, whilst the age-standardised data help to understand the trends due to factors other than demographic change. Data on absolute YLL provide information on the contribution of different causes to the number of deaths (with or without adjustment for changes in the underlying age structure of the population), whilst data on relative YLL provide information on the proportion of the total accounted for by each cause.

**Fig 1:**
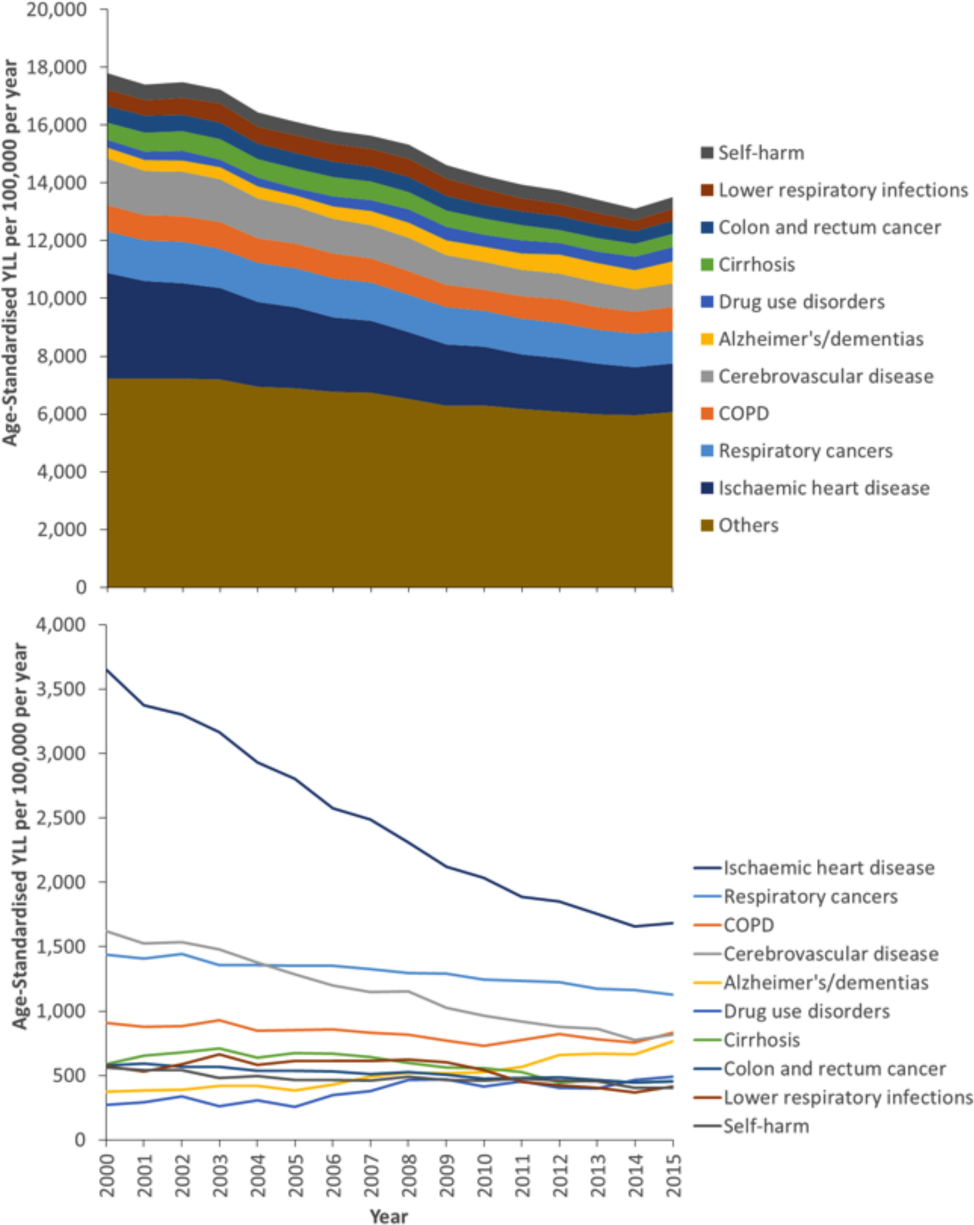
Temporal trends in the age-standardised YLL by cause (Scotland, 2000–2015, cumulative in upper panel, individual causes in lower panel).

**Fig 2:**
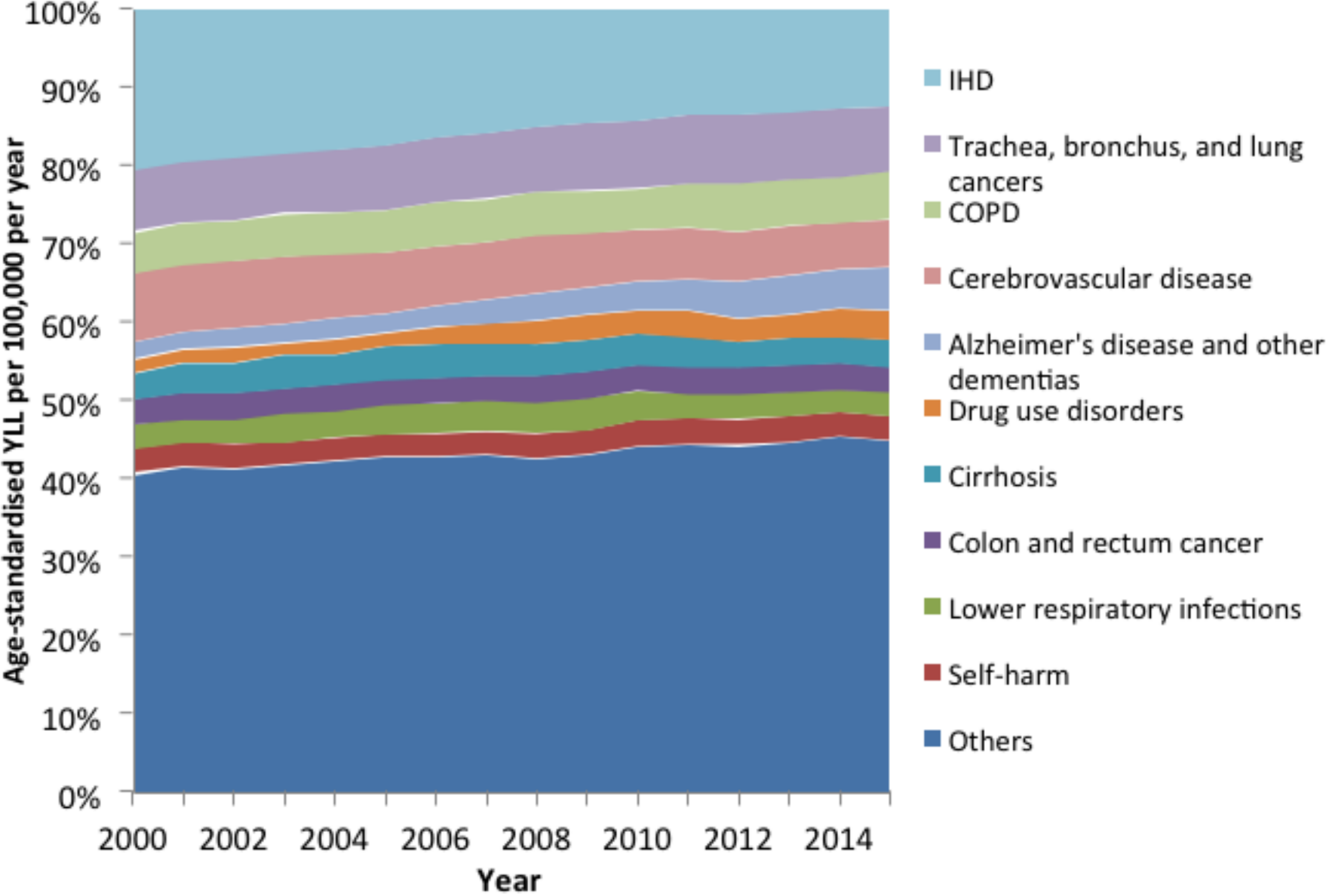
Percentage of total age-standardised YLL accounted for by specific causes (Scotland, 2000–2015).

Fig 1 shows that the ten leading causes of YLL accounted for around half of the total age-standardised YLL from 2000 to 2015. IHD accounted for the most YLL for the entire series, but there was a substantial reduction in the absolute (Fig 1) and relative (Fig 2) contribution (from 21% of the total to 12%) it made over time. Similar reductions were also seen for cerebrovascular disease. A smaller reduction was also seen over time for lower respiratory infections and self-harm. In contrast, drug use disorders and Alzheimer’s disease and other dementias, accounted for increases in absolute age-standardised YLL (of 389 and 221 per 100,000 per year respectively) and a substantially larger proportion of the total YLL over time (from 2% to 6% and from 2% to 4% respectively). COPD, cirrhosis, colon and rectum cancer and trachea, bronchus and lung cancers decreased in absolute terms and increased in relative terms over time (data for all causes, and separately for men and women, are provided in Table B in S5 File). As the data in Figs 1 and 2 are age-standardised the increases in causes such as Alzheimer’s Disease and other dementias cannot be due to ageing of the population. However, it could be that this represents ‘competing causes’ of death such that some of the averted deaths from the declining causes of death became deaths due to the increasing causes.

**Fig 3:**
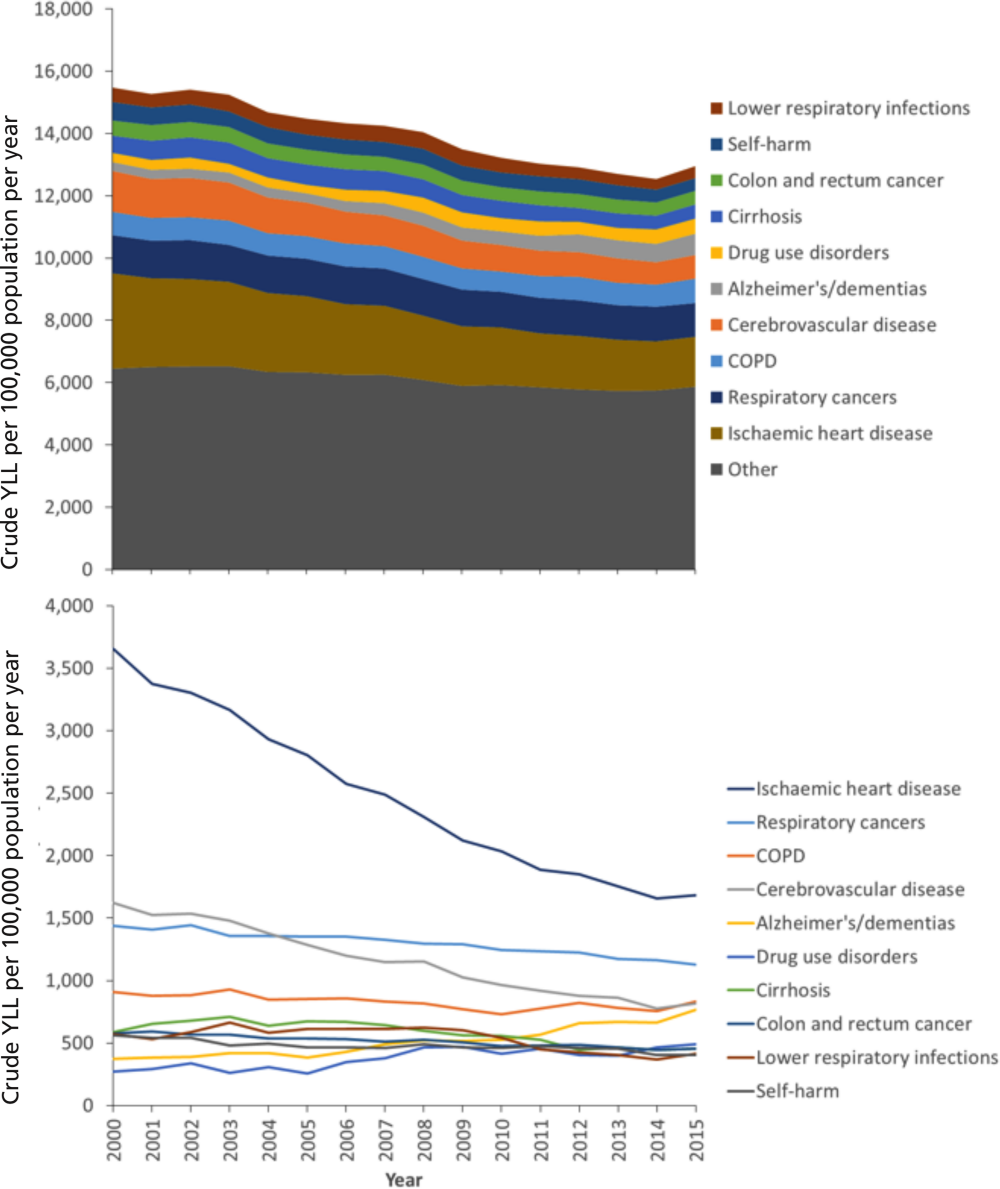
Temporal trends in the crude YLL per 100,000 population by cause (Scotland, 2000–2015, cumulative in upper panel, individual causes in lower panel).

**Fig 4:**
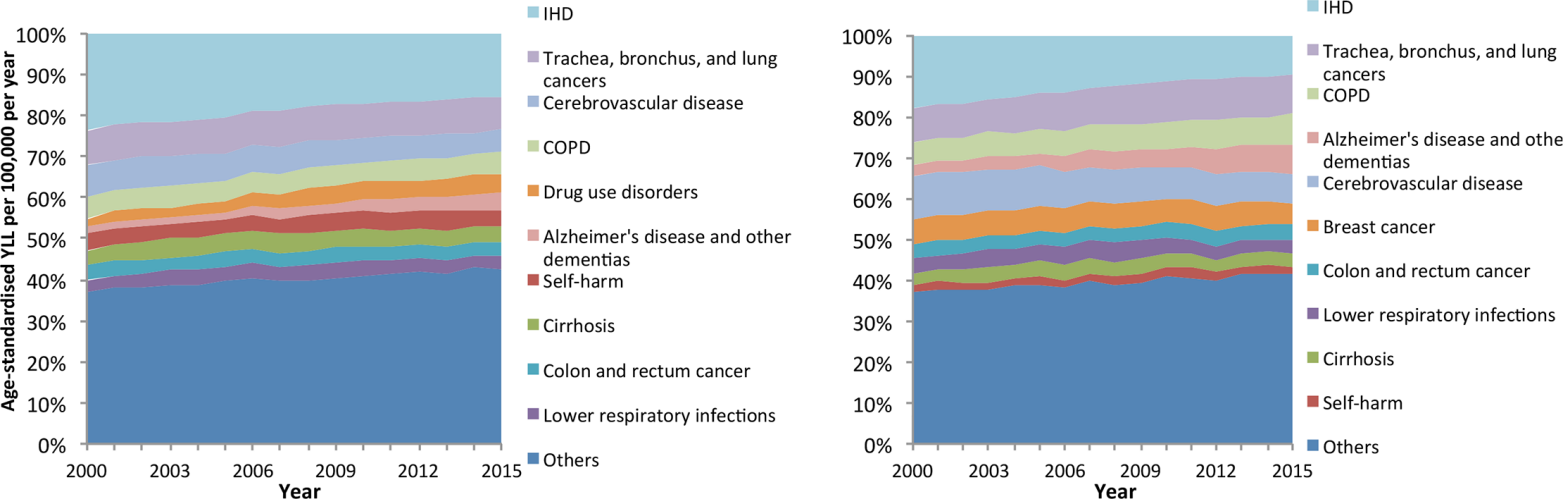
Percentage of total Age-Standardised YLL accounted for by specific causes (Scotland, 2000–2015).

Fig 3 provides the trends in crude YLL for the total population per 100,000 per year. The pattern is similar to that for the age-standardised rates, with declining YLL over time and just over half accounted for by the ten largest single contributing causes (Table C in S5 File). There is a ‘long tail’ of causes which account for a small number of YLL in Scotland, although the proportion of the YLL accounted for by this tail has increased over time as the total accounted for by several of the large contributors of YLL (particularly IHD and cerebrovascular disease) has declined. The YLL contributed by different causes varied slightly by sex with cirrhosis, drugs use and self-harm greater contributors in men, and breast cancer, lower respiratory tract infections greater contributors amongst women (Fig 4 and Table B in S5 File).

### Inequalities in years of life lost (YLL) by area deprivation

**Fig 5:**
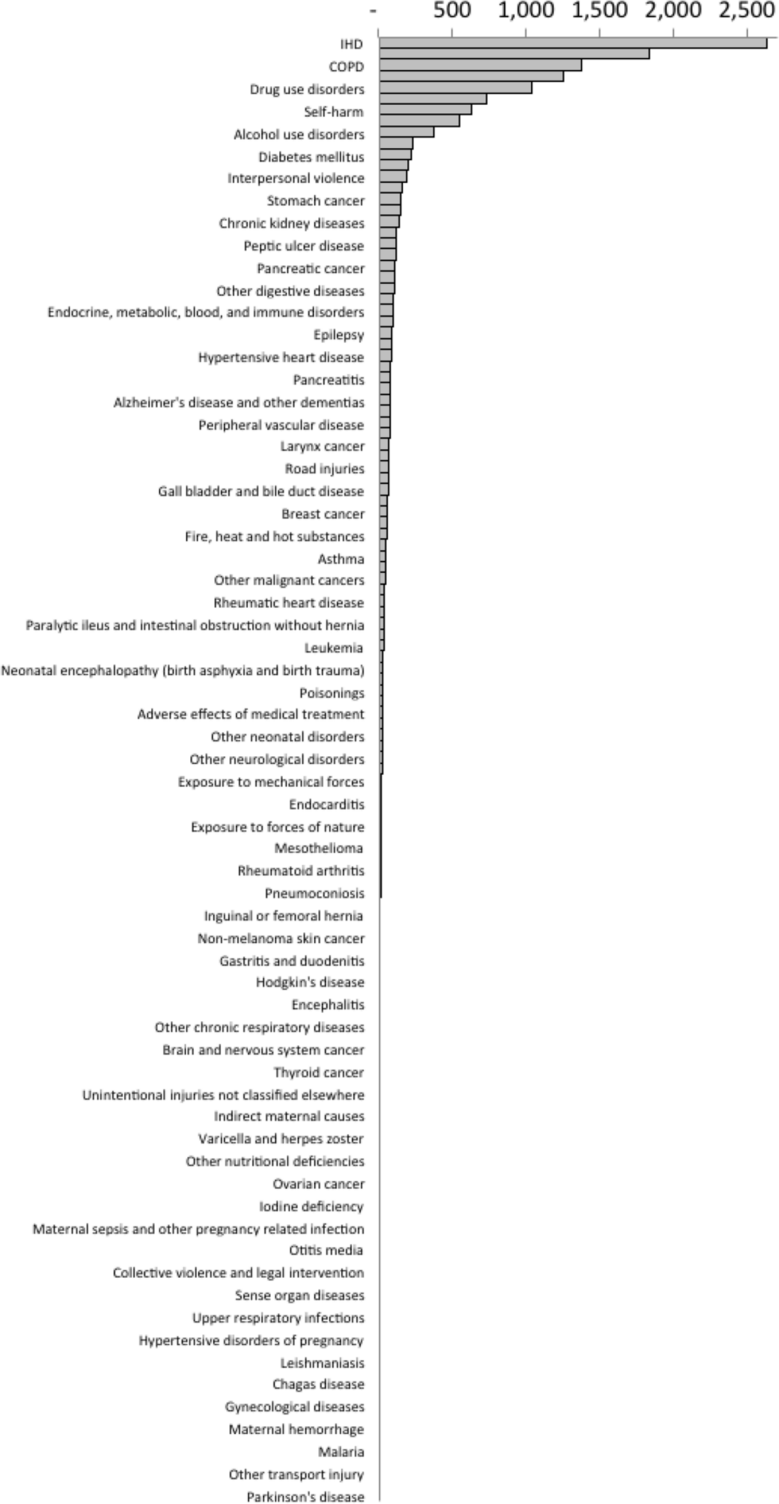
Mean slope index of inequality in age-standardised YLL per 100,000 population per year by cause of death (2001–2015).

For all-causes of YLL and the total population, the mean SII across deprivation deciles for the period 2001–2015 was 15,344 YLL, representing the difference in YLL across the population ranked by deprivation. The absolute inequalities were higher amongst men than women (18,688 and 12,133 YLL respectively). The inequalities were seen across most causes of death, but a substantial proportion of the all-cause mortality inequalities were accounted for by IHD, ‘trachea, bronchus, and lung cancers’, COPD, cirrhosis, drug use disorders, cerebrovascular disease, self-harm, lower respiratory infections, alcohol use disorders and other cardiovascular and circulatory diseases (Fig 5). For a small number of causes of YLL, the rates were higher in the least deprived areas (animal contact, other transport injury, malignant melanoma of skin, and Parkinson's disease) although the contributions to overall YLL due to these causes were very small.

**Fig 6:**
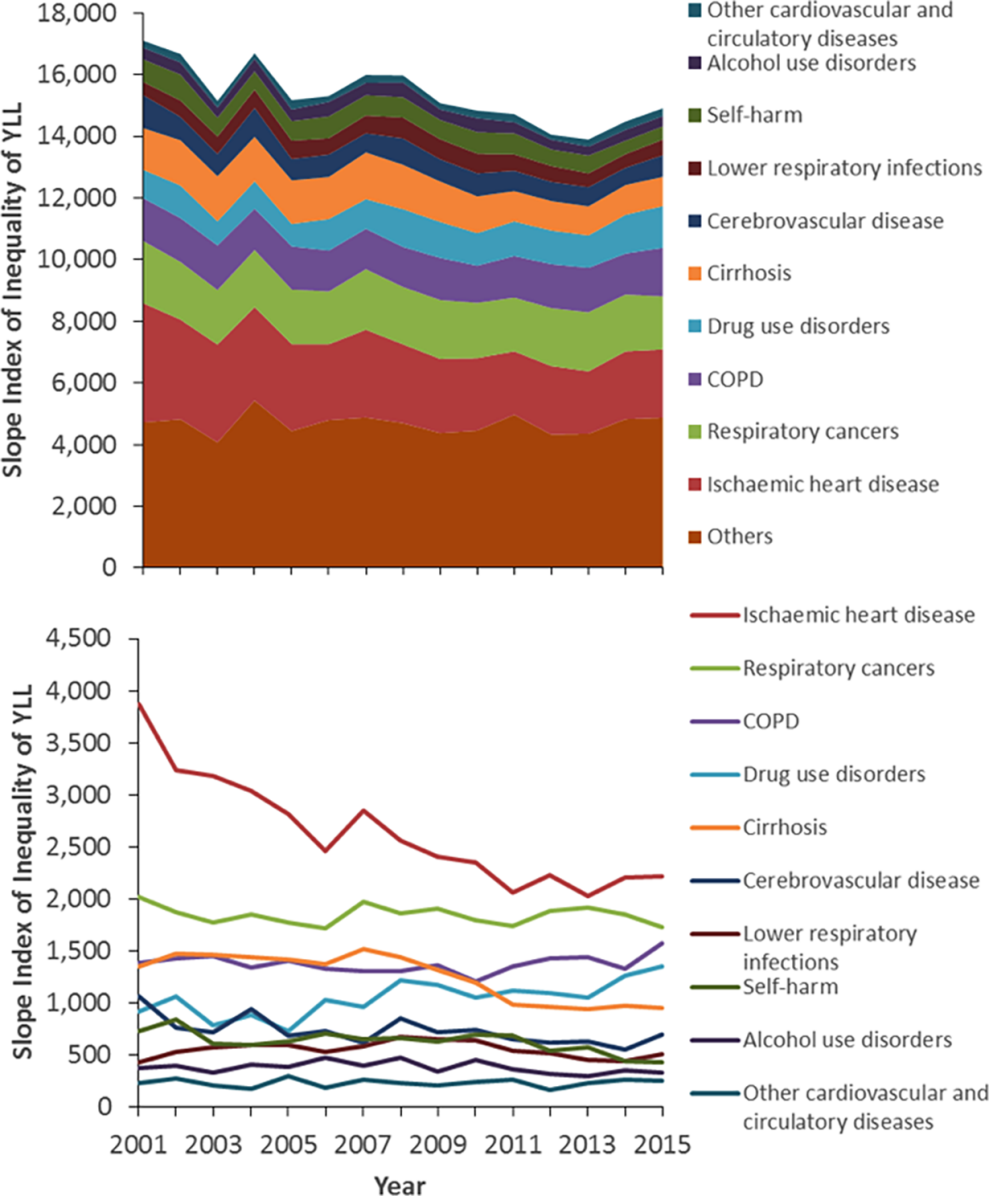
Trend in the slope index of inequality in age-standardised YLL per 100,000 population per year for the ten largest contributors of YLL (2001–2015, cumulative in upper panel, individual causes in lower panel).

Between 2001 and 2015, the absolute inequalities in YLL as measured by SII declined substantially from just over 17,000 YLL to just below 15,000 YLL (Fig 6). The declines were largely due to reduction in the inequalities in YLL due to IHD, cirrhosis, cerebrovascular disease and self-harm (alongside small contributions from a large number of causes) over the time period which were greater than the increases in inequalities seen for drug use disorders, urinary diseases, and Alzheimer’s disease and other dementias.

The decline in SII was more consistent over the time period for women than men, with a decline only evident for men after 2008. IHD is the greatest contributor to the SII for YLL in men, but only the third largest for women (after COPD and ‘trachea, bronchus and lung cancer’). Drug use disorders is the second largest contributor to the SII for men and the fourth for women. The decline in SII amongst women was almost entirely due to a large declines in inequality for IHD and small decreases for a wide range for the ‘other’ causes, as the SII increased for drug use disorders and Alzheimer’s and other dementias over the time period (S6 File).

**Fig 7:**
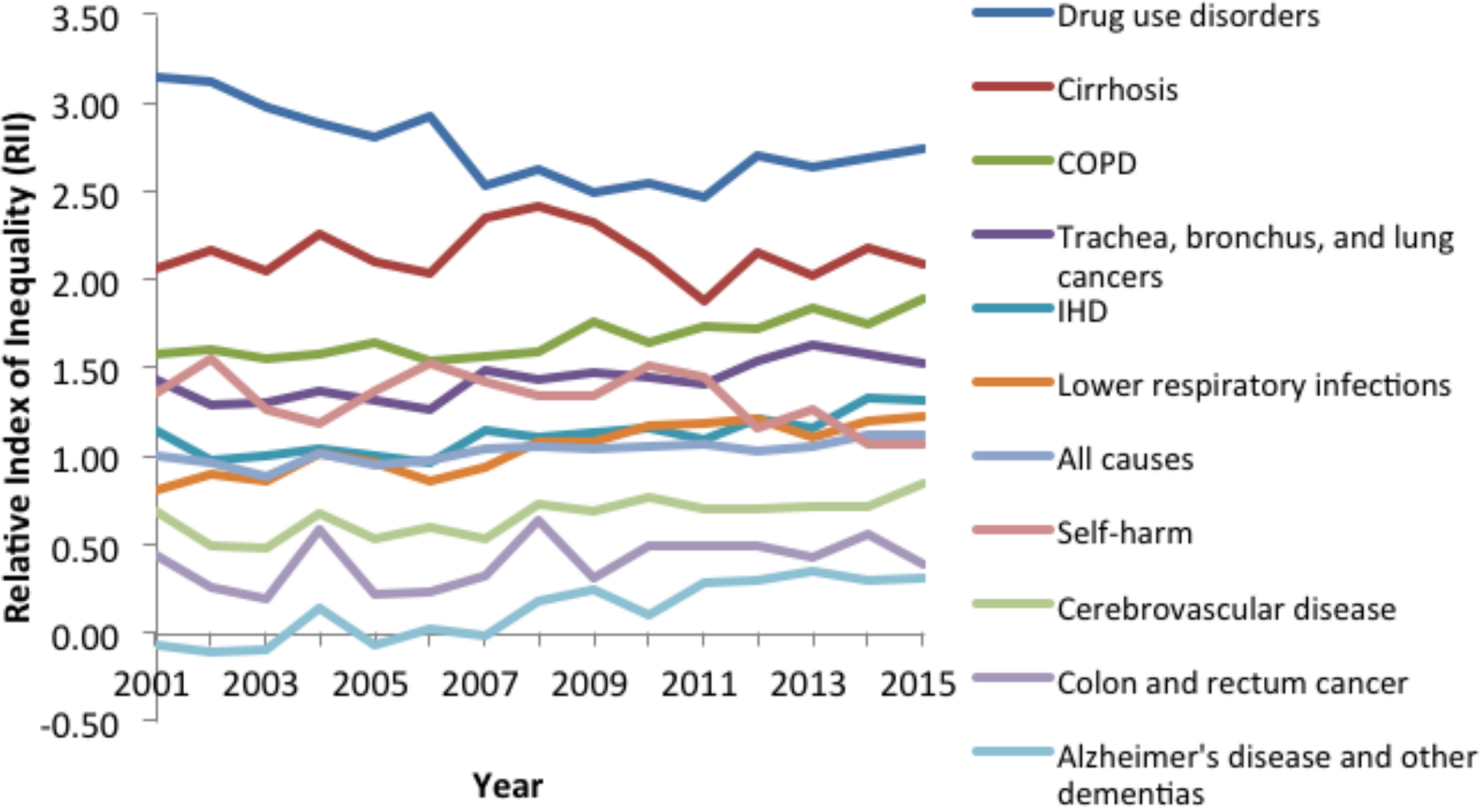
Trends in the relative index of inequality for the ten largest contributors to YLL (2001–2015).

Fig 7 details the trends in RII for the ten largest contributors to YLL for men and women combined. The RII for all causes of YLL steadily increased over the time series from 1.00 in 2001 to 1.11 by 2015 (Tables A-B in S6 File). For specific causes, the RIIs for drug use disorders were consistently highest from 2001–2015 amongst the diseases within the top 10 ranked by YLL. The RIIs were also particularly high for alcohol use disorders, cirrhosis and chronic obstructive pulmonary disease.

### Regional inequalities in years of life lost (YLL)

Greater Glasgow & Clyde, Lanarkshire and Ayrshire and Arran health board areas have the highest age-standardised mean YLL per 100,000 population over the period 2001–2015 in Scotland, with 17,805, 16,596 and 15,732 YLL respectively. Orkney and the Borders have the lowest mean YLL over this time period at 12,590 and 12,720 YLL. However, there is evidence of some convergence over time as the mean annual improvement in YLL in Greater Glasgow & Clyde was 418 YLL, the greatest reduction of all health board areas. The slowest mean annual reductions in YLL were in Shetland, Orkney and Dumfries and Galloway, areas with better than the Scottish mean YLL rates (Table A in S7 File).

## Discussion

### Main results

Between 2000 and 2015, between 10% and 12% of deaths were ill-defined. The proportion of deaths that were ill-defined slowly increased over time. The proportion of IDDs was higher amongst: women; amongst those aged 1–4 years, 25–34 years and >80 years; in more deprived areas; and in the island health boards. The total YLL fell from around 17,800 years per 100,000 population in 2000 to around 13,500 years by 2015. The largest individual causes of death that contributed to most to the overall burden of YLL were IHD, respiratory cancers, COPD, stroke and ‘Alzheimer’s disease and other dementias’. The proportion of total YLL due to IHD and stroke declined over time, but increased for Alzheimer’s and other dementias, and for drug use disorders. There were marked absolute inequalities in YLL by area deprivation, with a mean Slope Index of Inequality (SII) for all causes of 15,344 YLL between 2001 and 2015, with IHD and COPD the greatest contributors (which can be interpreted as a difference of this magnitude between the most and least deprived in the population based on the best fit line across groups ranked by deprivation). The Relative Index of Inequality (RII), which takes into account the mean YLL and therefore looks only at the ratio rather than the count difference, was highest for self-harm and lower respiratory infections.

### Strengths and weaknesses

This study has several important strengths. First, this study provides YLL data which focus attention on those deaths which lead to the greatest loss of lifespan and is a useful complement to widely available cause-specific mortality rates. This is the first publication of inequalities in YLL for Scotland, and the first showing inequalities by cause and over time. The SII indicates the scale of inequality in absolute terms for particular populations or causes and thus can help prioritise areas for policy action which are most responsible for shorter lives amongst people living in the most deprived areas. The RII indicates the inequalities as a ratio, and thereby highlights how many times worse it is for people living in more deprived circumstances and accounts for changes over time in the population overall.

We have redistributed the tenth of deaths in Scotland which are ill-defined to provide a more accurate classification of deaths in Scotland than is otherwise currently available. The data are presented as both crude rates to facilitate service planning decisions and age-standardised rates to facilitate comparisons within Scotland and between Scotland and other countries. The presentation of YLL by deprivation group and for sub-national areas within Scotland is also unique and can be used to inform prioritisation decisions for health and social policy. Finally, the study utilises deaths data which comprehensively cover the whole population and for which causes are determined by medical practitioners (and legal experts in the cases of some reportable-deaths such as suicide) which makes the quality of the data relatively good.

However, there are also a number of limitations. The grouping and nomenclature of some causes of death, although in line with the International Classification of Diseases, is somewhat arbitrary. This means that the sub-division of some categories into small strata makes the category appear less important than it might otherwise (e.g. IHD and other cardiovascular disease is split into different categories and the total contribution would have been much greater if these had been grouped). Some disease categories are also classified by their cause (e.g. alcohol use disorders) which is neither comprehensive (e.g. cirrhosis appears in another category) nor consistent with the categorisation of other causes of death. Some causes of death may also be under-recorded in some groups, as has been suspected in relation to people living with dementia who may have died due to influenza in 2015, but who were not recorded as such [12]. In a similar way, competing and contributory causes of death can be misrepresented in the mortality data, since only a single cause of death is represented in the data and the counterfactual situation (i.e. what someone would have died of if that particular cause had not been present) is unknown [13,14]. Although we have recoded IDDs, we are not clear what proportion of deaths are misclassified to well-defined causes on death certificates. We also used the redistribution algorithms used as part of the Global Burden of Disease project which may not be completely applicable to Scottish deaths data as it is based on global patterns of death certification practice and mortality epidemiology.

Our measure of deprivation is an area-based rather than individual measure and is therefore likely to misclassify many individuals into categories that do not reflect their individual experiences [15]. Furthermore, the SIMD deprivation measure includes a basket of weighted health measures in its derivation (thereby raising the theoretical possibility of reverse causality whereby people are ordered by the health outcome rather than socioeconomic deprivation). However, the employment-income deprivation index (i.e. excluding the health measures) is very highly correlated with the overall SIMD index and so this is unlikely to be have changed the results.

We used Scottish-specific life tables for men and women which made the arbitrary lower mortality limits more realistic for Scottish policymakers and allowed the use of sex-specific tables, but this limits the comparability of the results here to other countries. The results in this paper do not in themselves provide sufficient information for prioritisation of policy and practice as the full burden of disease (i.e. the combination of Years Lived with Disability (YLD) and YLL) and the Population Attributable Fractions (PAFs) for relevant exposures are required (alongside the evidence base for effective interventions). However, this study provides the substrate for that further work to be undertaken.

### How it fits with the rest of the literature

The redistribution of IDDs is a useful early step towards understanding the underlying causal processes leading to premature death, although not a substitute for better understanding, and recording of, the causes of death [16]. The use of YLL has the potential to give greater prominence to causes of death which are more common at younger ages (and which therefore generate a greater loss of potential years of life), but the relatively low number of such deaths in Scotland has meant that such causes are not substantial contributors to the overall YLL. As with any mortality measure, causes of ill-health which do not generally lead to death for most of the prevalent cases (e.g. mental ill-health, visual loss, hearing loss, back pain, etc.) do not feature prominently in the YLL causes list. The utility of this study is therefore to focus on the causes of death rather than the causes of overall burden of disease, but also to provide the substrate for further work to calculate the overall burden. Similar to other countries, we have found that overall age-standardised YLL is in general decline [16], that there are rising trends for some specific causes [17], and that inequalities on some measures are increasing [18].

### Implications

Efforts to improve our understanding of the causes of death, and to improve how deaths are certified, should continue in order to reduce the number of IDDs (and indeed misclassified deaths) in the future. This study, including the reclassification of IDDs and the calculation of YLL does provide one of the necessary components for the calculation of the full DALY for Scotland. The trends and causes of YLL, as well as the trends and causes of the inequalities in YLL, are similar to those for mortality and morbidity by other measures [19–21]. In that regard the policy implications remain similar to those already published. That means that policy efforts should be focused on: reducing inequalities in income, wealth and power; eradicating poverty; improving housing and the physical environment; increasing the availability and quality of work; and using legislation, regulation and taxation to reduce the adverse consequences of smoking, alcohol, illicit drugs and the food system [20,22].

## Conclusion

Between 10% and 12% of deaths in Scotland have ill-defined underlying causes, and these are most common at the extremes of age and for deaths between 25 and 34 years. The total number of YLL per 100,000 population in Scotland has declined from 2000 to 2015. The causes of death leading to most are the chronic diseases (IHD, COPD, stroke and cancers) alongside substance misuse, self-harm and increasingly Alzheimer’s disease and dementia. Inequalities in YLL, in both relative and absolute terms, are stark.

## Supporting information

S1 File Table A. Ill-defined death types and target diseases.
(DOCX)

S2 File Table A. Ill-defined deaths redistribution coefficients.
(XLSX)

S3 File Tables A-H. Ill-defined deaths by age, sex, time, SIMD and health board.
(XLSX)

S4 File Tables A-F. Ill-defined deaths by type.
(XLSX)

S5 File Table A. Crude and EASR YLL by time and cause.
(XLSX)

S6 File Tables A-B. EASR rates and YLL by SIMD.
(XLSX)

S7 File Table A. EASR rates and YLL by geography.
(XLSX)
