# Supplementary material for: Trends and inequalities in the burden of mortality in Scotland 2000–2015
Source: PLoS One. 2018 Aug 1;13(8):e0196906. doi: 10.1371/journal.pone.0196906 (PMC6070167; doi:10.1371/journal.pone.0196906)
Supplement: S1 File — Table A. Ill-defined death types and target diseases. (DOCX) [file pone.0196906.s001.docx]

**Table A - Redistribution of ill-defined deaths (IDD). Each IDD type is defined by a set of ICD-10 codes. An IDD is redistributed to all the target causes using either predetermined fixed coefficients or proportional allocation respecting the relative proportions of the target diseases within different gender and age groups.**

| **IDD Type** | **Method** | **IDD Description** | **ICD-10** | **Target Causes** |
| --- | --- | --- | --- | --- |
| interm-145 | Proportionally | Abdominal and pelvic pain | R10-R109 | Other cardiovascular and circulatory diseases, Peptic ulcer disease, Appendicitis, Paralytic ileus and intestinal obstruction without hernia, Gall bladder and bile duct disease, Pancreatitis, Other digestive diseases |
| interm-215 | Proportionally | Abnormalities of breathing and cyanosis | R06-R069 R230 | Tuberculosis, Lower respiratory infections, Preterm birth complications, Other neonatal disorders, Larynx cancer  Trachea, bronchus, and lung cancers, Mouth cancer, Cancer of other part of pharynx and oropharynx, Mesothelioma, Ischemic heart disease, Cerebrovascular disease, Hypertensive heart disease, Atrial fibrillation and flutter, Other cardiovascular and circulatory diseases, Chronic obstructive pulmonary disease, Pneumoconiosis,  Asthma, Interstitial lung disease and pulmonary sarcoidosis, Other chronic respiratory diseases, Chronic kidney diseases, Congenital anomalies, Poisonings, Adverse effects of medical treatment |
| interm-184 | Proportionally | Abscess of liver | K750 | Diarrheal diseases, Other digestive diseases |
| interm-209 | Proportionally | Abscess of lung and mediastinum | J85-J853 | Tuberculosis, Lower respiratory infections, Esophageal cancer, Trachea, bronchus, and lung cancers, Chronic obstructive pulmonary disease, Other chronic respiratory diseases, Alcohol use disorders |
| p099 | Proportionally | Accidental poisoning by and exposure to narcotics and psychodysleptics [hallucinogens], not elsewhere classified | X41-X429 | Drug use disorders, Poisonings |
| p100 | Proportionally | Accidental poisoning by and exposure to other and unspecified drugs, medicaments and biological substances | X44-X449 | Drug use disorders, Poisonings |
| interm-54 | Fixed | Acidosis | E872 | Lower respiratory infections, Chronic obstructive pulmonary disease, Cirrhosis, Diabetes mellitus, Chronic kidney diseases |
| interm-140 | Fixed | Acute and unspecified renal failure | N17-N179 N19-N199 | HIV/AIDS, Diarrheal diseases, Intestinal infectious diseases, Lower respiratory infections, Otitis media, Meningitis, Encephalitis, Measles, Varicella and herpes zoster, Maternal haemorrhage, Maternal sepsis and other pregnancy related infection, Obstructed labour, Maternal abortive outcome, Other maternal disorders, Sexually transmitted diseases excluding HIV, Other infectious diseases, Stomach cancer, Liver cancer, Colon and rectum cancer, Gallbladder and biliary tract cancer, Pancreatic cancer, Hodgkin's disease, Non-Hodgkin lymphoma, Multiple myeloma, Leukaemia, Other malignant cancers, Rheumatic heart disease, Ischemic heart disease, Hypertensive heart disease, Cardiomyopathy and myocarditis, Endocarditis, Other cardiovascular and circulatory diseases, Chronic obstructive pulmonary disease, Pneumoconiosis, Interstitial lung disease and pulmonary sarcoidosis, Cirrhosis, Appendicitis, Gall bladder and bile duct disease, Pancreatitis, Other digestive diseases, Eating disorders, Diabetes mellitus, Acute glomerulonephritis, Chronic kidney diseases, Urinary diseases and male infertility, Gynaecological diseases, Endocrine, metabolic, blood, and immune disorders, Other musculoskeletal disorders, Congenital anomalies, Skin and subcutaneous diseases, Sense organ diseases, Road injuries, Other transport injury, Falls, Drowning, Fire, heat and hot substances, Exposure to mechanical forces, Adverse effects of medical treatment, Animal contact, Foreign body, Unintentional injuries not classified elsewhere, Self-harm, Interpersonal violence, Exposure to forces of nature, Collective violence and legal intervention |
| p101 | Proportionally | Acute nasopharyngitis, pharyngitis, tonsillitis, laryngitis and tracheitis, supraglottitis, epiglottitis, epiglottitis, | J00-J000 J02 J028-J03 J038-J04 J041-J043 J051-J069 | Lower respiratory infections, Upper respiratory infections |
| interm-104 | Proportionally | Amyloidosis | E853 E855-E857 | Tuberculosis, HIV/AIDS, Diarrheal diseases, Intestinal infectious diseases, Lower respiratory infections, Upper respiratory infections, Otitis media, Meningitis, Encephalitis, Varicella and herpes zoster, Malaria, Other neglected tropical diseases, Protein-energy malnutrition, Iodine deficiency, Iron-deficiency anaemia, Other nutritional deficiencies, Sexually transmitted diseases excluding HIV, Hepatitis, Other infectious diseases, Esophageal cancer, Stomach cancer, Liver cancer, Larynx cancer, Trachea, bronchus, and lung cancers, Breast cancer, Cervical cancer, Uterine cancer, Prostate cancer, Colon and rectum cancer, Mouth cancer, Nasopharynx cancer, Cancer of other part of pharynx and oropharynx, Gallbladder and biliary tract cancer, Pancreatic cancer, Malignant melanoma of skin, Non-melanoma skin cancer, Ovarian cancer, Testicular cancer, Kidney and other urinary organ cancers, Bladder cancer, Brain and nervous system cancer, Thyroid cancer, Mesothelioma, Hodgkin's disease, Non-Hodgkin lymphoma, Multiple myeloma, Leukaemia, Other malignant cancers, Other non-malignant neoplasms, Rheumatic heart disease, Ischemic heart disease, Cerebrovascular disease, Hypertensive heart disease, Cardiomyopathy and myocarditis, Atrial fibrillation and flutter, Aortic aneurysm, Peripheral vascular disease, Endocarditis, Other cardiovascular and circulatory diseases, Chronic obstructive pulmonary disease, Pneumoconiosis, Asthma, Interstitial lung disease and pulmonary sarcoidosis, Other chronic respiratory diseases, Cirrhosis, Peptic ulcer disease, Gastritis and duodenitis, Appendicitis, Paralytic ileus and intestinal obstruction without hernia, Inguinal or femoral hernia, Non-infective inflammatory bowel disease, Vascular disorders of intestine, Gall bladder and bile duct disease, Pancreatitis, Other digestive diseases, Alzheimer's disease and other dementias, Parkinson's disease, Epilepsy, Multiple sclerosis, Other neurological disorders, Schizophrenia, Alcohol use disorders, Drug use disorders, Eating disorders, Diabetes mellitus, Acute glomerulonephritis, Chronic kidney diseases, Urinary diseases and male infertility, Gynaecological diseases, Hemoglobinopathies and Haemolytic anaemias, Endocrine, metabolic, blood, and immune disorders, Rheumatoid arthritis, Other musculoskeletal disorders, Congenital anomalies, Skin and subcutaneous diseases, Sense organ diseases, Road injuries, Other transport injury, Falls, Drowning, Fire, heat and hot substances, Poisonings, Exposure to mechanical forces, Adverse effects of medical treatment, Animal contact, Foreign body, Unintentional injuries not classified elsewhere, Self-harm, Interpersonal violence, Exposure to forces of nature |
| interm-72 | Fixed | Anoxic brain damage, not elsewhere classified. Benign intracranial hypertension. Hypertensive encephalopathy | G931-G932 I674 | Ischemic heart disease, Cerebrovascular disease, Drug use disorders, Drowning, Foreign body, Unintentional injuries not classified elsewhere |
| interm-216 | Proportionally | Asphyxia and hypoxemia | R092 | Tuberculosis, Lower respiratory infections, Larynx cancer, Trachea, bronchus, and lung cancers, Cancer of other part of pharynx and oropharynx, Other malignant cancers, Other non-malignant neoplasms, Rheumatic heart disease, Ischemic heart disease, Cerebrovascular disease, Hypertensive heart disease, Cardiomyopathy and myocarditis, Atrial fibrillation and flutter, Peripheral vascular disease, Endocarditis, Other cardiovascular and circulatory diseases, Chronic obstructive pulmonary disease, Pneumoconiosis, Asthma, Interstitial lung disease and pulmonary sarcoidosis, Other chronic respiratory diseases, Alzheimer's disease and other dementias, Epilepsy, Alcohol use disorders, Drug use disorders, Chronic kidney diseases, Endocrine, metabolic, blood, and immune disorders, Congenital anomalies, Road injuries, Falls, Drowning, Poisonings, Exposure to mechanical forces, Adverse effects of medical treatment, Foreign body, Unintentional injuries not classified elsewhere, Self-harm, Interpersonal violence |
| interm-79 | Fixed | Atherosclerosis | I70-I701 I709 | Ischemic heart disease, Cerebrovascular disease, Hypertensive heart disease, Aortic aneurysm, Other cardiovascular and circulatory diseases, Chronic kidney diseases |
| interm-74 | Fixed | Atrioventricular and left bundle-branch block. Other conduction disorders | I44-I459 | Chagas disease, Ischemic heart disease, Cardiomyopathy and myocarditis, Endocrine, metabolic, blood, and immune disorders, Congenital anomalies, Adverse effects of medical treatment |
| interm-152 | Fixed | Cachexia , underweight | R634 R636 R64 | Drowning, Fire, heat and hot substances, Poisonings, Exposure to mechanical forces, Adverse effects of medical treatment, Animal contact, Foreign body |
| interm-208 | Proportionally | Cardiac arrest. Hypotension. Nonspecific low blood-pressure reading. Syncope and collapse. Shock, not elsewhere classifi | I46-I469 I95-I951 I958-I959 R031 R55 R57 R578 | Tuberculosis, Diarrheal diseases, Lower respiratory infections, Meningitis, Encephalitis, Neonatal encephalopathy (birth asphyxia and birth trauma), Protein-energy malnutrition, Iron-deficiency anaemia, Other infectious diseases, Esophageal cancer, Stomach cancer, Liver cancer, Trachea, bronchus, and lung cancers, Breast cancer, Cervical cancer, Uterine cancer, Prostate cancer, Colon and rectum cancer, Mouth cancer, Nasopharynx cancer, Cancer of other part of pharynx and oropharynx, Gallbladder and biliary tract cancer, Pancreatic cancer, Malignant melanoma of skin, Ovarian cancer, Testicular cancer, Kidney and other urinary organ cancers, Bladder cancer, Brain and nervous system cancer, Thyroid cancer, Mesothelioma, Hodgkin's disease, Non-Hodgkin lymphoma, Multiple myeloma, Leukaemia, Other malignant cancers, Rheumatic heart disease, Ischemic heart disease, Cerebrovascular disease, Hypertensive heart disease, Cardiomyopathy and myocarditis, Atrial fibrillation and flutter, Aortic aneurysm, Peripheral vascular disease, Endocarditis, Other cardiovascular and circulatory diseases, Chronic obstructive pulmonary disease, Asthma, Interstitial lung disease and pulmonary sarcoidosis, Other chronic respiratory diseases, Cirrhosis, Appendicitis, Paralytic ileus and intestinal obstruction without hernia, Non-infective inflammatory bowel disease, Vascular disorders of intestine, Gall bladder and bile duct disease, Pancreatitis, Other digestive diseases, Alzheimer's disease and other dementias, Epilepsy, Schizophrenia, Alcohol use disorders, Drug use disorders, Diabetes mellitus, Chronic kidney diseases, Hemoglobinopathies and Haemolytic anaemias, Endocrine, metabolic, blood, and immune disorders, Congenital anomalies, Skin and subcutaneous diseases, Road injuries, Falls, Poisonings, Exposure to mechanical forces, Adverse effects of medical treatment, Foreign body, Unintentional injuries not classified elsewhere, Self-harm, Interpersonal violence |
| interm-150 | Proportionally | Cardiogenic shock | R570 | Rheumatic heart disease, Ischemic heart disease, Hypertensive heart disease, Aortic aneurysm, Other cardiovascular and circulatory diseases, Chronic kidney diseases |
| interm-71 | Fixed | Cerebral oedema | G936 | Meningitis, Encephalitis, Brain and nervous system cancer, Ischemic heart disease, Cerebrovascular disease, Hypertensive heart disease, Drug use disorders, Diabetes mellitus, Chronic kidney diseases, Endocrine, metabolic, blood, and immune disorders, Adverse effects of medical treatment, Unintentional injuries not classified elsewhere, Self-harm, Interpersonal violence |
| interm-117 | Proportionally | Cerebral palsy. Spastic hemiplegia, paraplegia, tetraplegia and other paralytic syndromes | G80-G809 G821 G824 G830 G838 | Tuberculosis, HIV/AIDS, Diarrheal diseases, Intestinal infectious diseases, Lower respiratory infections, Upper respiratory infections, Otitis media, Meningitis, Encephalitis, Varicella and herpes zoster, Malaria, Cysticercosis, Other neglected tropical diseases, Maternal haemorrhage, Maternal sepsis and other pregnancy related infection, Hypertensive disorders of pregnancy, Obstructed labour, Maternal abortive outcome, Indirect maternal causes, Other maternal disorders, Preterm birth complications, Neonatal encephalopathy (birth asphyxia and birth trauma), Other neonatal disorders, Protein-energy malnutrition, Iodine deficiency, Iron-deficiency anaemia, Other nutritional deficiencies, Sexually transmitted diseases excluding HIV, Hepatitis, Other infectious diseases, Esophageal cancer, Stomach cancer, Liver cancer, Larynx cancer, Trachea, bronchus, and lung cancers, Breast cancer, Cervical cancer, Uterine cancer, Prostate cancer, Colon and rectum cancer, Mouth cancer, Nasopharynx cancer, Cancer of other part of pharynx and oropharynx, Gallbladder and biliary tract cancer, Pancreatic cancer, Malignant melanoma of skin, Non-melanoma skin cancer, Ovarian cancer, Testicular cancer, Kidney and other urinary organ cancers, Bladder cancer, Brain and nervous system cancer, Thyroid cancer, Mesothelioma, Hodgkin's disease, Non-Hodgkin lymphoma, Multiple myeloma, Leukaemia, Other malignant cancers, Other non-malignant neoplasms, Rheumatic heart disease, Ischemic heart disease, Cerebrovascular disease, Hypertensive heart disease, Cardiomyopathy and myocarditis, Atrial fibrillation and flutter, Aortic aneurysm, Peripheral vascular disease, Endocarditis, Other cardiovascular and circulatory diseases, Chronic obstructive pulmonary disease, Pneumoconiosis, Asthma, Interstitial lung disease and pulmonary sarcoidosis, Other chronic respiratory diseases, Cirrhosis, Peptic ulcer disease, Gastritis and duodenitis, Appendicitis, Paralytic ileus and intestinal obstruction without hernia, Inguinal or femoral hernia, Non-infective inflammatory bowel disease, Vascular disorders of intestine, Gall bladder and bile duct disease, Pancreatitis, Other digestive diseases, Alzheimer's disease and other dementias, Parkinson's disease, Epilepsy, Multiple sclerosis, Other neurological disorders, Schizophrenia, Alcohol use disorders, Drug use disorders, Eating disorders, Diabetes mellitus, Acute glomerulonephritis, Chronic kidney diseases, Urinary diseases and male infertility, Gynaecological diseases, Hemoglobinopathies and Haemolytic anaemias, Endocrine, metabolic, blood, and immune disorders, Rheumatoid arthritis, Other musculoskeletal disorders, Congenital anomalies, Skin and subcutaneous diseases, Sense organ diseases, Road injuries, Other transport injury, Falls, Drowning, Fire, heat and hot substances, Poisonings, Exposure to mechanical forces, Adverse effects of medical treatment, Animal contact, Foreign body, Unintentional injuries not classified elsewhere, Self-harm, Interpersonal violence, Exposure to forces of nature, Collective violence and legal intervention |
| interm-65 | Fixed | Chronic respiratory failure | J961-J968 | Tuberculosis, Rheumatic heart disease, Ischemic heart disease, Cardiomyopathy and myocarditis, Other cardiovascular and circulatory diseases, Chronic obstructive pulmonary disease, Pneumoconiosis, Asthma, Other chronic respiratory diseases |
| p027 | Proportionally | Complications and ill-defined descriptions of heart disease | I51 I518-I519 | Iodine deficiency, Iron-deficiency anaemia, Rheumatic heart disease, Ischemic heart disease, Hypertensive heart disease, Cardiomyopathy and myocarditis, Atrial fibrillation and flutter, Endocarditis, Other cardiovascular and circulatory diseases, Chronic obstructive pulmonary disease, Pneumoconiosis, Hemoglobinopathies and Haemolytic anaemias, Endocrine, metabolic, blood, and immune disorders, Congenital anomalies, Road injuries, Falls, Exposure to mechanical forces, Self-harm, Interpersonal violence, Forces of nature, war, and legal intervention |
| interm-120 | Fixed | Compression of brain | G935 | Meningitis, Encephalitis, Brain and nervous system cancer, Cerebrovascular disease, Road injuries, Falls, Exposure to mechanical forces, Self-harm, Interpersonal violence, Forces of nature, war, and legal intervention |
| p042 | Proportionally | Condition originating in the perinatal period, unspecified | P06 P16-P18 P30-P342 P40-P49 P62-P69 P73 P79 P82 P85-P89 P969-P999 | Lower respiratory infections, Varicella and herpes zoster, Preterm birth complications, Neonatal encephalopathy (birth asphyxia and birth trauma), Sepsis and other infectious disorders of the newborn baby, Haemolytic disease in foetus and newborn and other neonatal jaundice, Other neonatal disorders, Other infectious diseases, Congenital anomalies, Stillbirth |
| p070 | Proportionally | Contact with sharp object, undetermined intent | Y274-Y275 Y28-Y282 Y284 Y286-Y289 | Exposure to mechanical forces, Self-harm, Interpersonal violence |
| interm-148 | Proportionally | Convulsions, not elsewhere classified | R56 R561-R569 | Collective violence and legal intervention, Exposure to forces of nature, Cerebrovascular disease, Diabetes mellitus, Endocrine, metabolic, blood, and immune disorders, Meningitis, Encephalitis, Epilepsy, Brain and nervous system cancer, Road injuries, Falls, Drowning, Exposure to mechanical forces, Self-harm |
| p074 | Proportionally | Crashing of motor vehicle, undetermined intent | Y32-Y329 | Road injuries, Self-harm, Interpersonal violence |
| p017 | Proportionally | Disease of blood and blood-forming organs, unspecified | D759 D79 D85 D87-D88 D90-D99 | HIV/AIDS, Iron-deficiency anaemia, Other nutritional deficiencies, Interstitial lung disease and pulmonary sarcoidosis, Hemoglobinopathies and Haemolytic anaemias, Endocrine, metabolic, blood, and immune disorders |
| p040 | Proportionally | Disorder of urinary system, unspecified | N399 | Tuberculosis, HIV/AIDS, Diarrheal diseases, Intestinal infectious diseases, Lower respiratory infections, Otitis media, Meningitis, Encephalitis, Whooping cough, Measles, Varicella and herpes zoster, Chagas disease, Food-borne trematodiases, Leishmaniasis, Yellow fever, Other neglected tropical diseases, Maternal sepsis and other pregnancy related infection, Indirect maternal causes, Neonatal encephalopathy (birth asphyxia and birth trauma), Protein-energy malnutrition, Iodine deficiency, Iron-deficiency anaemia, Other nutritional deficiencies, Sexually transmitted diseases excluding HIV, Hepatitis, Other infectious diseases, Esophageal cancer, Stomach cancer, Liver cancer, Larynx cancer, Trachea, bronchus, and lung cancers, Breast cancer, Cervical cancer, Uterine cancer, Colon and rectum cancer, Mouth cancer, Nasopharynx cancer, Cancer of other part of pharynx and oropharynx, Gallbladder and biliary tract cancer, Pancreatic cancer, Malignant melanoma of skin, Non-melanoma skin cancer, Ovarian cancer, Kidney and other urinary organ cancers, Bladder cancer, Brain and nervous system cancer, Thyroid cancer, Mesothelioma, Hodgkin's disease, Non-Hodgkin lymphoma, Multiple myeloma, Leukaemia, Other malignant cancers, Other non-malignant neoplasms, Rheumatic heart disease, Ischemic heart disease, Cerebrovascular disease, Hypertensive heart disease, Cardiomyopathy and myocarditis, Atrial fibrillation and flutter, Aortic aneurysm, Peripheral vascular disease, Endocarditis, Other cardiovascular and circulatory diseases, Chronic obstructive pulmonary disease, Pneumoconiosis, Asthma, Interstitial lung disease and pulmonary sarcoidosis, Other chronic respiratory diseases, Cirrhosis, Peptic ulcer disease, Gastritis and duodenitis, Appendicitis, Paralytic ileus and intestinal obstruction without hernia, Inguinal or femoral hernia, Non-infective inflammatory bowel disease, Vascular disorders of intestine, Gall bladder and bile duct disease, Pancreatitis, Other digestive diseases, Alzheimer's disease and other dementias, Parkinson's disease, Epilepsy, Multiple sclerosis, Other neurological disorders, Schizophrenia, Alcohol use disorders, Drug use disorders, Eating disorders, Diabetes mellitus, Acute glomerulonephritis, Chronic kidney diseases, Urinary diseases and male infertility, Gynaecological diseases, Hemoglobinopathies and Haemolytic anaemias, Endocrine, metabolic, blood, and immune disorders, Rheumatoid arthritis, Other musculoskeletal disorders, Congenital anomalies, Skin and subcutaneous diseases, Sense organ diseases, Road injuries, Other transport injury, Falls, Drowning, Fire, heat and hot substances, Poisonings, Exposure to mechanical forces, Adverse effects of medical treatment, Animal contact, Foreign body, Unintentional injuries not classified elsewhere, Self-harm, Interpersonal violence, Forces of nature, war, and legal intervention, Exposure to forces of nature, Collective violence and legal intervention |
| p063 | Proportionally | Drowning and submersion, undetermined intent | Y21-Y219 | Drowning, Self-harm, Interpersonal violence |
| interm-70 | Fixed | Encephalopathy, unspecified | G934 | Lower respiratory infections, Encephalitis, Neonatal encephalopathy (birth asphyxia and birth trauma), Haemolytic disease in foetus and newborn and other neonatal jaundice, Other nutritional deficiencies, Cerebrovascular disease, Cirrhosis, Alzheimer's disease and other dementias, Other neurological disorders, Alcohol use disorders |
| p020 | Proportionally | Endocrine disorder, unspecified | E17-E19 E349-E358 | Diabetes mellitus, Chronic kidney diseases, Endocrine, metabolic, blood, and immune disorders |
| p082 | Proportionally | Endocrine gland, unspecified | C759 D44 D449 D497 | Thyroid cancer, Other malignant cancers |
| interm-213 | Proportionally | Excessive, frequent and irregular menstruation, other abnormal uterine and vaginal bleeding, pain associated with female | N92-N949 N950 | Tuberculosis, Maternal haemorrhage, Obstructed labour, Maternal abortive outcome, Other maternal disorders, Iron-deficiency anaemia, Other nutritional deficiencies, Sexually transmitted diseases excluding HIV, Other infectious diseases, Cervical cancer, Uterine cancer, Ovarian cancer, Gynaecological diseases, Hemoglobinopathies and Haemolytic anaemias, Endocrine, metabolic, blood, and immune disorders, Falls, Adverse effects of medical treatment, Unintentional injuries not classified elsewhere |
| p068 | Proportionally | Exposure to smoke, fire and flames, undetermined intent | Y26-Y262 Y264-Y269 | Fire, heat and hot substances, Self-harm, Interpersonal violence |
| p046 | Proportionally | Exposure to unspecified factor | W47-W48 W63 W71-W72 W82 W95-W97 W98 X07 X55-X56 X59-X599 | Alcohol use disorders, Drug use disorders, Road injuries, Other transport injury, Falls, Drowning, Fire, heat and hot substances, Poisonings, Exposure to mechanical forces, Animal contact, Foreign body, Unintentional injuries not classified elsewhere, Exposure to forces of nature |
| p072 | Proportionally | Falling, jumping or pushed from a high place, undetermined intent | Y283 Y285 Y291-Y309 | Falls, Self-harm, Interpersonal violence |
| p073 | Proportionally | Falling, lying or running before or into moving object, undetermined intent | Y31-Y319 | Road injuries, Other transport injury, Self-harm |
| p079 | Proportionally | Female genital organ, unspecified | C579 C59 D073 D28 D289 D39-D390 D399 N842-N848 | Cervical cancer, Uterine cancer, Ovarian cancer, Other malignant cancers |
| interm-115 | Fixed | Flaccid hemiplegia | G81-G819 | Meningitis, Encephalitis, Brain and nervous system cancer, Cerebrovascular disease, Diabetes mellitus, Road injuries, Falls, Exposure to mechanical forces, Collective violence and legal intervention |
| interm-110 | Proportionally | Fluid overload | E877 | Iron-deficiency anaemia, Rheumatic heart disease, Ischemic heart disease, Hypertensive heart disease, Cardiomyopathy and myocarditis, Chronic obstructive pulmonary disease, Chronic kidney diseases, Congenital anomalies |
| interm-194 | Fixed | Haematemesis, melaena and gastrointestinal haemorrhage, unspecified | K920-K922 | Stomach cancer, Colon and rectum cancer, Other malignant cancers, Cirrhosis, Peptic ulcer disease, Other digestive diseases |
| p005 | Proportionally | Haemophilus influenzae infection, unspecified | A492 | Lower respiratory infections, Upper respiratory infections, Other musculoskeletal disorders, Skin and subcutaneous diseases |
| interm-143 | Fixed | Haemorrhage from respiratory passages | R04 R041-R049 | Tuberculosis, Lower respiratory infections, Other infectious diseases, Trachea, bronchus, and lung cancers, Chronic obstructive pulmonary disease |
| interm-149 | Fixed | Haemorrhage, not elsewhere classified | R571-R574 R58 | Maternal haemorrhage, Obstructed labour, Maternal abortive outcome, Other maternal disorders, Peptic ulcer disease, Other digestive diseases, Road injuries, Falls, Exposure to mechanical forces, Self-harm, Interpersonal violence, Exposure to forces of nature, Collective violence and legal intervention |
| interm-128 | Fixed | Haemothorax | I312-I314 J942 | Road injuries, Falls, Exposure to mechanical forces, Self-harm, Interpersonal violence, Forces of nature, war, and legal intervention |
| p062 | Proportionally | Hanging, strangulation and suffocation, undetermined intent | Y20-Y209 | Exposure to mechanical forces, Self-harm, Interpersonal violence |
| interm-76 | Proportionally | Heart failure, cardiomegaly, other pulmonary heart diseases, disease of pulmonary vessels, unspecified, systolic (congestive) heart failure. Diseminated intravascular coagulation | I27-I270 I272-I279 I289 I50-I509 I517 | Iodine deficiency, Iron-deficiency anaemia, Rheumatic heart disease, Ischemic heart disease, Hypertensive heart disease, Cardiomyopathy and myocarditis, Endocarditis, Other cardiovascular and circulatory diseases, Chronic obstructive pulmonary disease, Pneumoconiosis, Hemoglobinopathies and Haemolytic anaemias, Endocrine, metabolic, blood, and immune disorders, Congenital anomalies |
| interm-61 | Fixed | Hepatic failure, not elsewhere classified | K72-K720 | Food-borne trematodiases, Leishmaniasis, Maternal sepsis and other pregnancy related infection, Hepatitis, Cirrhosis, Gall bladder and bile duct disease, Other digestive diseases, Drug use disorders, Hemoglobinopathies and Haemolytic anaemias, Endocrine, metabolic, blood, and immune disorders, Poisonings, Adverse effects of medical treatment |
| interm-218 | Proportionally | Hepatomegaly and splenomegaly, not elsewhere classified, unspecified jaundice | R16-R170 | Cirrhosis, Congenital anomalies, Hypertensive heart disease, Ischemic heart disease, Diabetes mellitus, Diarrheal diseases, Gall bladder and bile duct disease, Other digestive diseases, Pancreatitis,  Hemoglobinopathies and Haemolytic anaemias, Falls, Poisonings, Self-harm, Lower respiratory infections, Alcohol use disorders, Drug use disorders, Breast cancer, Colon and rectum cancer, Leukaemia, Liver cancer, Non-Hodgkin lymphoma, Other non-malignant neoplasms, Other malignant cancers, Pancreatic cancer, Sepsis and other infectious disorders of the newborn baby, Chagas disease, Leishmaniasis, Iron-deficiency anaemia, Other chronic respiratory diseases, Tuberculosis, Other infectious diseases, Gallbladder and biliary tract cancer, Stomach cancer, Other neonatal disorders |
| interm-118 | Fixed | Hydrocephalus | G91-G912 G914-G919 | Meningitis, Encephalitis, Other neglected tropical diseases, Preterm birth complications, Neonatal encephalopathy (birth asphyxia and birth trauma), Other neonatal disorders, Brain and nervous system cancer, Congenital anomalies, Road injuries, Falls, Exposure to mechanical forces, Self-harm, Interpersonal violence, Forces of nature, war, and legal intervention |
| interm-109 | Fixed | Hyperkalaemia | E875 | Chronic kidney diseases, Adverse effects of medical treatment |
| interm-206 | Proportionally | Hyperosmolality and hypernatremia, amnestic disorder due to known physiological condition, delirium due to known physiol | E870 F04-F059 | Diarrheal diseases, Protein-energy malnutrition, Cerebrovascular disease, Alzheimer's disease and other dementias, Schizophrenia, Alcohol use disorders, Drug use disorders, Eating disorders, Diabetes mellitus, Endocrine, metabolic, blood, and immune disorders, Adverse effects of medical treatment, Unintentional injuries not classified elsewhere, Exposure to forces of nature |
| interm-80 | Fixed | Hypertension, abnormal bood-preassure, epistaxis | I10-I109 I15-I159 R03-R030 R040 | Ischemic heart disease, Cerebrovascular disease, Hypertensive heart disease, Aortic aneurysm, Other cardiovascular and circulatory diseases, Chronic kidney diseases |
| interm-107 | Fixed | Hypokalemia | E876 | Diarrheal diseases, Paralytic ileus and intestinal obstruction without hernia, Eating disorders, Diabetes mellitus, Endocrine, metabolic, blood, and immune disorders |
| p044 | Proportionally | Ill-defined causes in F07, G91, H26, N12, M19-M21, Y33, Y86-Y99 | F072 G913 M120 M125 M191-M219 M872 S00-T983 Y33-Y349 Y86-Y87 Y872 Y89 Y899-Y999 | Alcohol use disorders, Drug use disorders, Road injuries, Other transport injury, Falls, Drowning, Fire, heat and hot substances, Poisonings, Exposure to mechanical forces, Adverse effects of medical treatment, Animal contact, Foreign body, Unintentional injuries not classified elsewhere, Self-harm, Interpersonal violence, Exposure to forces of nature |
| interm-207 | Proportionally | Intracranial and intraspinal abscess and granuloma, Intracranial and intraspinal phlebitis and thrombophlebitis | G06-G080 G930 | Otitis media, Sexually transmitted diseases excluding HIV, Endocarditis, Other chronic respiratory diseases, Diabetes mellitus, Endocrine, metabolic, blood, and immune disorders, Skin and subcutaneous diseases, Sense organ diseases, Adverse effects of medical treatment |
| p087 | Proportionally | Kaposi's sarcoma | C46-C469 | HIV/AIDS, Other malignant cancers |
| p080 | Proportionally | Male genital organ, unspecified | C639 D076 D29 D299 D40 D409 | Prostate cancer, Testicular cancer, Other malignant cancers |
| p083 | Proportionally | Malignant neoplasm of other and ill- defined sites | C42 C76 C764-C77 C773-C774 C778-C78 C79 C792-C809 C87 C98-C99 D08-D09 D099 D360 D369 D48 D487-D49 D498-D499 D54 | Esophageal cancer, Stomach cancer, Liver cancer, Larynx cancer, Trachea, bronchus, and lung cancers, Breast cancer, Cervical cancer, Uterine cancer, Prostate cancer, Colon and rectum cancer, Mouth cancer, Nasopharynx cancer, Cancer of other part of pharynx and oropharynx, Gallbladder and biliary tract cancer, Pancreatic cancer, Malignant melanoma of skin, Non-melanoma skin cancer, Ovarian cancer, Testicular cancer, Kidney and other urinary organ cancers, Bladder cancer, Brain and nervous system cancer, Thyroid cancer, Mesothelioma, Hodgkin's disease, Non-Hodgkin lymphoma, Multiple myeloma, Leukaemia, Other malignant cancers |
| p084 | Proportionally | Malignant neoplasm of other and ill- defined sites | C760-C761 C770-C771 C780-C783 D17-D219 | Esophageal cancer, Larynx cancer, Trachea, bronchus, and lung cancers, Breast cancer, Mouth cancer, Nasopharynx cancer, Cancer of other part of pharynx and oropharynx, Malignant melanoma of skin, Non-melanoma skin cancer, Brain and nervous system cancer, Thyroid cancer, Mesothelioma, Other malignant cancers |
| p075 | Proportionally | Malignant neoplasm of other and ill-defined sites in the lip, oral cavity and pharynx | C14-C149 D000 D10 D109 D370 | Mouth cancer, Nasopharynx cancer, Cancer of other part of pharynx and oropharynx |
| p077 | Proportionally | Malignant neoplasm of other and ill-defined sites in the respiratory system and intrathoracic organs | C39-C399 D02 D024-D029 D14 D144 D38 D386 D491 | Larynx cancer, Trachea, bronchus, and lung cancers, Mesothelioma, Other malignant cancers |
| p081 | Proportionally | Malignant neoplasm of unspecified urinary organs | C68 C689 D091 D30 D309 D41 D419 | Kidney and other urinary organ cancers, Bladder cancer, Other malignant cancers |
| p078 | Proportionally | Malignant neoplasm of uterus, part unspecified | C55-C559 D26 D267-D269 | Cervical cancer, Uterine cancer |
| p085 | Proportionally | Malignant neoplasms of ill-defined, secondary and unspecified sites in adboment, pelvis or small intestine | C762-C763 C772 C775 C784-C788 C790-C791 | Esophageal cancer, Stomach cancer, Liver cancer, Colon and rectum cancer, Gallbladder and biliary tract cancer, Pancreatic cancer, Other malignant cancers |
| p048 | Proportionally | Motor- or nonmotor-vehicle accident, type of vehicle unspecified | V89-V899 | Road injuries, Other transport injury |
| interm-210 | Proportionally | Nausea and vomiting | R11-R112 | Cirrhosis, Chronic kidney diseases, Congenital anomalies, Atrial fibrillation and flutter, Cardiomyopathy and myocarditis, Hypertensive heart disease, Ischemic heart disease,  Peripheral vascular disease, Cerebrovascular disease, Diabetes mellitus, Diarrheal diseases, Appendicitis, Gall bladder and bile duct disease, Other digestive diseases, Gastritis and duodenitis,  Non-infective inflammatory bowel disease, Paralytic ileus and intestinal obstruction without hernia,  Pancreatitis, Vascular disorders of intestine, Endocrine, metabolic, blood, and immune disorders, Falls,  Adverse effects of medical treatment, Poisonings, Lower respiratory infections, Meningitis, Alcohol use disorders, Drug use disorders, Brain and nervous system cancer, Colon and rectum cancer, Esophageal cancer, Gallbladder and biliary tract cancer, Liver cancer, Non-Hodgkin lymphoma, Mouth cancer, Nasopharynx cancer, Other malignant cancers, Cancer of other part of pharynx and oropharynx, Pancreatic cancer, Haemolytic disease in foetus and newborn and other neonatal jaundice, Preterm birth complications, Alzheimer's disease and other dementias, Epilepsy, Parkinson's disease, Protein-energy malnutrition, Chronic obstructive pulmonary disease, Urinary diseases and male infertility |
| p076 | Proportionally | Neoplasm and carcinoma of other and ill-defined digestive organs | C26-C29 C35-C36 D00 D01 D014-D019 D13 D139 D37 D376-D379 D490 | Esophageal cancer, Stomach cancer, Liver cancer, Colon and rectum cancer, Gallbladder and biliary tract cancer, Pancreatic cancer, Other malignant cancers |
| p041 | Proportionally | Obstetric death of unspecified cause | O17-O19 O27 O37-O39 O49-O59 O78-O79 O93-O959 | Maternal haemorrhage, Maternal sepsis and other pregnancy related infection, Hypertensive disorders of pregnancy, Obstructed labour, Maternal abortive outcome, Indirect maternal causes, Other maternal disorders |
| interm-211 | Proportionally | Obstructive and reflux uropathy. Hematuria | N13-N139 R31-R319 | Tuberculosis, Kidney and other urinary organ cancers, Non-Hodgkin lymphoma, Multiple myeloma, Leukaemia, Diabetes mellitus, Chronic kidney diseases, Urinary diseases and male infertility, Congenital anomalies, Road injuries, Other transport injury, Falls, Drowning, Fire, heat and hot substances, Poisonings, Exposure to mechanical forces, Adverse effects of medical treatment, Animal contact, Foreign body, Unintentional injuries not classified elsewhere, Self-harm, Interpersonal violence, Exposure to forces of nature, Collective violence and legal intervention |
| interm-105 | Fixed | Organ-limited amyloidosis | E854 E858-E859 | Tuberculosis, Other infectious diseases, Kidney and other urinary organ cancers, Multiple myeloma, Chronic obstructive pulmonary disease, Non-infective inflammatory bowel disease, Alzheimer's disease and other dementias, Parkinson's disease, Other neurological disorders, Chronic kidney diseases, Endocrine, metabolic, blood, and immune disorders, Rheumatoid arthritis |
| p001 | Proportionally | Other and unspecified bacterial and infectious diseases. Endocrine, nutritional and metabolic diseases. Mental and Mental and behavioural disorders | A59-A599 A71-A719 A740 B07-B09 B30-B309 B319 B323-B324 B35-B369 B85-B854 B87-B889 B940 E50-E509 F063-F064 F09-F099 F17-F179 F30-F49 F51-F990 G15-G19 G27-G29 G32-G34 G38-G39 G42-G448 G47-G472 G474-G609 G62-G69 G74-G79 G84-G894 G94-G942 G99-G990 G992-H05 H052-H580 H588-H598 H60-H699 H71-H99 K00-K19 K23-K24 K30 K319 L20-L309 L40-L45 L49-L509 L52-L540 L55-L609 L62-L689 L70-L879 L90-L929 L94-L959 L985-L998 M04 M10-M12 M122-M124 M128-M190 M22-M29 M37-M39 M432-M49 M492-M64 M651-M71 M712-M73 M738-M859 M873-M879 M891-M894 M90-M999 N09 N24 N328-N338 N35-N359 N37-N38 N393-N398 N40-N409 N42-N434 N46-N489 N52-N649 N66-N69 N78-N79 N84 N849-N86 N88-N915 N95 N951-N959 N97-N979 Q08-Q103 Q19 Q29 Q360-Q369 Q46-Q49 Q88 Q94 R070 R08-R09 R093 R12-R159 R19-R196 R198-R23 R231-R309 R32-R399 R41-R499 R51-R538 R540-R549 R550 R576 R580-R633 R635 R638 R640-R651 R66-R729 R74-R78 R786-R948 R950-R999 U049 | Tuberculosis, HIV/AIDS, Diarrheal diseases, Intestinal infectious diseases, Lower respiratory infections, Upper respiratory infections, Otitis media, Meningitis, Encephalitis, Whooping cough, Measles, Varicella and herpes zoster, Malaria, Chagas disease, Food-borne trematodiases, Leishmaniasis, Cysticercosis, Yellow fever, Other neglected tropical diseases, Maternal haemorrhage, Maternal sepsis and other pregnancy related infection, Hypertensive disorders of pregnancy, Obstructed labour, Maternal abortive outcome, Indirect maternal causes, Other maternal disorders, Preterm birth complications, Neonatal encephalopathy (birth asphyxia and birth trauma), Sepsis and other infectious disorders of the newborn baby, Haemolytic disease in foetus and newborn and other neonatal jaundice, Other neonatal disorders, Protein-energy malnutrition, Iodine deficiency, Iron-deficiency anaemia, Other nutritional deficiencies, Sexually transmitted diseases excluding HIV, Hepatitis, Other infectious diseases, Esophageal cancer, Stomach cancer, Liver cancer, Larynx cancer, Trachea, bronchus, and lung cancers, Breast cancer, Cervical cancer, Uterine cancer, Prostate cancer, Colon and rectum cancer, Mouth cancer, Nasopharynx cancer, Cancer of other part of pharynx and oropharynx, Gallbladder and biliary tract cancer, Pancreatic cancer, Malignant melanoma of skin, Non-melanoma skin cancer, Ovarian cancer, Testicular cancer, Kidney and other urinary organ cancers, Bladder cancer, Brain and nervous system cancer, Thyroid cancer, Mesothelioma, Hodgkin's disease, Non-Hodgkin lymphoma, Multiple myeloma, Leukaemia, Other malignant cancers, Other non-malignant neoplasms, Rheumatic heart disease, Ischemic heart disease, Cerebrovascular disease, Hypertensive heart disease, Cardiomyopathy and myocarditis, Atrial fibrillation and flutter, Aortic aneurysm, Peripheral vascular disease, Endocarditis, Other cardiovascular and circulatory diseases, Chronic obstructive pulmonary disease, Pneumoconiosis, Asthma, Interstitial lung disease and pulmonary sarcoidosis, Other chronic respiratory diseases, Cirrhosis, Peptic ulcer disease, Gastritis and duodenitis, Appendicitis, Paralytic ileus and intestinal obstruction without hernia, Inguinal or femoral hernia, Non-infective inflammatory bowel disease, Vascular disorders of intestine, Gall bladder and bile duct disease, Pancreatitis, Other digestive diseases, Alzheimer's disease and other dementias, Parkinson's disease, Epilepsy, Multiple sclerosis, Other neurological disorders, Schizophrenia, Alcohol use disorders, Drug use disorders, Eating disorders, Diabetes mellitus, Acute glomerulonephritis, Chronic kidney diseases, Urinary diseases and male infertility, Gynaecological diseases, Hemoglobinopathies and Haemolytic anaemias, Endocrine, metabolic, blood, and immune disorders, Rheumatoid arthritis, Other musculoskeletal disorders, Congenital anomalies, Skin and subcutaneous diseases, Sudden infant death syndrome, Sense organ diseases, Road injuries, Other transport injury, Falls, Drowning, Fire, heat and hot substances, Poisonings, Exposure to mechanical forces, Adverse effects of medical treatment, Animal contact, Foreign body, Unintentional injuries not classified elsewhere, Self-harm, Interpersonal violence, Forces of nature, war, and legal intervention, Exposure to forces of nature, Collective violence and legal intervention, Stillbirth |
| p091 | Proportionally | Other and unspecified disorders of circulatory system | I99 I998-I999 | Iodine deficiency, Iron-deficiency anaemia, Rheumatic heart disease, Ischemic heart disease, Cerebrovascular disease, Hypertensive heart disease, Cardiomyopathy and myocarditis, Atrial fibrillation and flutter, Aortic aneurysm, Peripheral vascular disease, Endocarditis, Other cardiovascular and circulatory diseases, Chronic obstructive pulmonary disease, Pneumoconiosis, Cirrhosis, Other digestive diseases, Chronic kidney diseases, Hemoglobinopathies and Haemolytic anaemias, Endocrine, metabolic, blood, and immune disorders, Congenital anomalies, Road injuries, Falls, Exposure to mechanical forces, Self-harm, Interpersonal violence, Forces of nature, war, and legal intervention |
| p066 | Proportionally | Other and unspecified firearm discharge, undetermined intent | Y233 Y238-Y249 | Exposure to mechanical forces, Self-harm, Interpersonal violence |
| p004 | Proportionally | Other bacterial diseases or bacterial infection, not elsewhere classified | A48 A488-A49 A493-A499 | Tuberculosis, Diarrheal diseases, Intestinal infectious diseases, Other infectious diseases |
| interm-73 | Fixed | Other cardiac arrhythmias | I49-I499 | Chagas disease, Ischemic heart disease, Cardiomyopathy and myocarditis, Endocrine, metabolic, blood, and immune disorders, Adverse effects of medical treatment |
| p094 | Proportionally | Other diseases of bronchus, not elsewhere classified | J98-J980 J984-J986 | Chronic obstructive pulmonary disease, Pneumoconiosis, Asthma, Interstitial lung disease and pulmonary sarcoidosis, Other chronic respiratory diseases |
| p095 | Proportionally | Other diseases of intestine | K63-K634 K638-K639 | Gastritis and duodenitis, Appendicitis, Paralytic ileus and intestinal obstruction without hernia, Non-infective inflammatory bowel disease, Vascular disorders of intestine, Gall bladder and bile duct disease, Pancreatitis, Other digestive diseases |
| p090 | Proportionally | Other disorders of brain | G938-G939 G96-G969 G98-G989 G991 | Meningitis, Encephalitis, Cerebrovascular disease, Alzheimer's disease and other dementias, Parkinson's disease, Epilepsy, Multiple sclerosis, Other neurological disorders, Endocrine, metabolic, blood, and immune disorders |
| interm-55 | Fixed | Other disorders of fluid, electrolyte and acid-base balance | E87 E874 E878-E879 | Diarrheal diseases, Lower respiratory infections, Chronic obstructive pulmonary disease, Asthma, Cirrhosis, Paralytic ileus and intestinal obstruction without hernia, Eating disorders, Diabetes mellitus, Chronic kidney diseases, Endocrine, metabolic, blood, and immune disorders, Fire, heat and hot substances, Unintentional injuries not classified elsewhere, Exposure to forces of nature |
| p096 | Proportionally | Other immunodeficiencies | D84 D849 D898-D899 | HIV/AIDS, Endocrine, metabolic, blood, and immune disorders |
| interm-185 | Proportionally | Other inflammatory liver diseases | K751 | Intestinal infectious diseases, Diarrheal diseases, Non-infective inflammatory bowel disease,  Vascular disorders of intestine, Other digestive diseases, Food-borne trematodiases, Leishmaniasis |
| p022 | Proportionally | Other mental disorders due to brain damage and dysfunction and to physical disease | F06-F061 F065-F070 F078-F08 | Meningitis, Encephalitis, Sexually transmitted diseases excluding HIV, Brain and nervous system cancer, Alzheimer's disease and other dementias, Parkinson's disease, Multiple sclerosis, Other neurological disorders, Alcohol use disorders, Drug use disorders, Poisonings |
| p092 | Proportionally | Other specified respiratory disorders | J988-J998 | Lower respiratory infections, Upper respiratory infections, Chronic obstructive pulmonary disease, Pneumoconiosis, Asthma, Interstitial lung disease and pulmonary sarcoidosis, Other chronic respiratory diseases |
| interm-219 | Fixed | Paraplegia (paraparesis) and quadriplegia (quadriparesis) | G82-G820 G822-G823 G825-G829 G834-G835 G839 R18-R189 | Tuberculosis, Brain and nervous system cancer, Other malignant cancers, Road injuries, Falls, Exposure to mechanical forces, Self-harm, Interpersonal violence, Forces of nature, war, and legal intervention |
| interm-59 | Fixed | Peritonitis | K65-K661 K669 | Cirrhosis, Peptic ulcer disease, Appendicitis, Paralytic ileus and intestinal obstruction without hernia, Inguinal or femoral hernia, Non-infective inflammatory bowel disease, Gall bladder and bile duct disease, Pancreatitis, Other digestive diseases, Urinary diseases and male infertility, Endocrine, metabolic, blood, and immune disorders, Road injuries, Other transport injury, Drowning, Exposure to mechanical forces, Adverse effects of medical treatment, Animal contact, Foreign body, Unintentional injuries not classified elsewhere, Self-harm, Interpersonal violence |
| interm-66 | Fixed | Pleural effusion, not elsewhere classified, Other pleural conditions | J90-J900 J94-J941 J948-J949 R091 | Tuberculosis, Lower respiratory infections, Trachea, bronchus, and lung cancers, Breast cancer, Mesothelioma, Non-Hodgkin lymphoma, Other malignant cancers, Rheumatic heart disease, Ischemic heart disease, Cardiomyopathy and myocarditis, Other cardiovascular and circulatory diseases, Chronic obstructive pulmonary disease, Pneumoconiosis, Cirrhosis, Rheumatoid arthritis, Other musculoskeletal disorders, Falls, Drowning, Fire, heat and hot substances, Poisonings, Exposure to mechanical forces, Adverse effects of medical treatment, Animal contact, Foreign body, Unintentional injuries not classified elsewhere |
| interm-187 | Fixed | Pneumonitis due to solids and liquids | J69-J699 | Other infectious diseases, Cerebrovascular disease, Chronic obstructive pulmonary disease, Parkinson's disease, Epilepsy, Exposure to mechanical forces, Foreign body |
| interm-127 | Fixed | Pneumothorax | J93-J931 J938-J939 | Tuberculosis, Lower respiratory infections, Whooping cough, Trachea, bronchus, and lung cancers, Chronic obstructive pulmonary disease, Asthma, Interstitial lung disease and pulmonary sarcoidosis, Endocrine, metabolic, blood, and immune disorders, Congenital anomalies, Road injuries, Falls, Exposure to mechanical forces, Self-harm, Interpersonal violence, Collective violence and legal intervention |
| p053 | Proportionally | Poisoning by and exposure to antiepileptic, sedative-hypnotic, antiparkinsonism and psychotropic drugs, not elsewhere cl | Y11-Y119 | Drug use disorders, Poisonings, Self-harm, Interpersonal violence |
| p054 | Proportionally | Poisoning by and exposure to narcotics and psychodysleptics [hallucinogens], not elsewhere classified, undetermined inte | Y12-Y129 | Drug use disorders, Poisonings, Self-harm, Interpersonal violence |
| p052 | Proportionally | Poisoning by and exposure to nonopioid analgesics, antipyretics and antirheumatics, undetermined intent | Y10-Y109 | Poisonings, Self-harm, Interpersonal violence |
| p058 | Proportionally | Poisoning by and exposure to organic solvents and halogenated hydrocarbons and their vapours, undetermined intent | Y16-Y169 | Poisonings, Self-harm |
| p061 | Proportionally | Poisoning by and exposure to other and unspecified chemicals and noxious substances, undetermined intent | Y19-Y199 | Drug use disorders, Self-harm |
| p056 | Proportionally | Poisoning by and exposure to other and unspecified drugs, medicaments and biological substances, undetermined intent | Y14-Y149 | Drug use disorders, Poisonings, Self-harm, Interpersonal violence |
| p055 | Proportionally | Poisoning by and exposure to other drugs acting on the autonomic nervous system, undetermined intent | Y13-Y139 | Drug use disorders, Poisonings, Self-harm, Interpersonal violence |
| p059 | Proportionally | Poisoning by and exposure to other gases and vapours, undetermined intent | Y17-Y179 | Poisonings, Self-harm |
| p060 | Proportionally | Poisoning by and exposure to pesticides, undetermined intent | Y18-Y189 | Drug use disorders, Poisonings, Self-harm |
| interm-132 | Fixed | Pulmonary collapse | J981 | Lower respiratory infections, Trachea, bronchus, and lung cancers, Rheumatic heart disease, Ischemic heart disease, Cardiomyopathy and myocarditis, Other cardiovascular and circulatory diseases, Chronic obstructive pulmonary disease, Pneumoconiosis, Cirrhosis, Chronic kidney diseases, Foreign body |
| interm-63 | Fixed | Pulmonary embolism | I26-I269 | Maternal haemorrhage, Obstructed labour, Maternal abortive outcome, Other maternal disorders, Endocarditis, Other cardiovascular and circulatory diseases, Road injuries, Other transport injury, Falls, Drowning |
| interm-75 | Fixed | Pulmonary oedema | J81-J811 | Rheumatic heart disease, Ischemic heart disease, Hypertensive heart disease, Cardiomyopathy and myocarditis, Other cardiovascular and circulatory diseases, Chronic obstructive pulmonary disease, Chronic kidney diseases |
| interm-67 | Fixed | Pyothorax without fistula | J86-J869 | Tuberculosis, Rheumatic heart disease, Ischemic heart disease, Cardiomyopathy and myocarditis, Other cardiovascular and circulatory diseases, Chronic obstructive pulmonary disease, Pneumoconiosis, Asthma, Other chronic respiratory diseases |
| interm-64 | Fixed | Respiratory failure, not elsewhere classified, compensatory emphysema and acute respiratory distress syndrome | J80-J809 J96-J960 J969 J983 | Tuberculosis, Lower respiratory infections, Whooping cough, Rheumatic heart disease, Ischemic heart disease, Cardiomyopathy and myocarditis, Other cardiovascular and circulatory diseases, Chronic obstructive pulmonary disease, Pneumoconiosis, Asthma, Other chronic respiratory diseases, Drug use disorders, Diabetes mellitus, Endocrine, metabolic, blood, and immune disorders, Poisonings, Interpersonal violence |
| p065 | Proportionally | Rifle, shotgun and larger firearm discharge, undetermined intent | Y23-Y232 Y234-Y237 | Exposure to mechanical forces, Self-harm, Interpersonal violence |
| p002 | Proportionally | Senility | R54 | Tuberculosis, HIV/AIDS, Diarrheal diseases, Lower respiratory infections, Encephalitis, Other neglected tropical diseases, Protein-energy malnutrition, Hepatitis, Other infectious diseases, Esophageal cancer, Stomach cancer, Liver cancer, Larynx cancer, Trachea, bronchus, and lung cancers, Breast cancer, Cervical cancer, Uterine cancer, Prostate cancer, Colon and rectum cancer, Mouth cancer, Nasopharynx cancer, Cancer of other part of pharynx and oropharynx, Gallbladder and biliary tract cancer, Pancreatic cancer, Malignant melanoma of skin, Non-melanoma skin cancer, Ovarian cancer, Testicular cancer, Kidney and other urinary organ cancers, Bladder cancer, Brain and nervous system cancer, Thyroid cancer, Mesothelioma, Hodgkin's disease, Non-Hodgkin lymphoma, Multiple myeloma, Leukaemia, Other malignant cancers, Other non-malignant neoplasms, Rheumatic heart disease, Ischemic heart disease, Cerebrovascular disease, Hypertensive heart disease, Cardiomyopathy and myocarditis, Atrial fibrillation and flutter, Aortic aneurysm, Peripheral vascular disease, Endocarditis, Other cardiovascular and circulatory diseases, Chronic obstructive pulmonary disease, Pneumoconiosis, Asthma, Interstitial lung disease and pulmonary sarcoidosis, Other chronic respiratory diseases, Cirrhosis, Peptic ulcer disease, Gastritis and duodenitis, Paralytic ileus and intestinal obstruction without hernia, Inguinal or femoral hernia, Non-infective inflammatory bowel disease, Vascular disorders of intestine, Gall bladder and bile duct disease, Pancreatitis, Other digestive diseases, Alzheimer's disease and other dementias, Parkinson's disease, Epilepsy, Multiple sclerosis, Other neurological disorders, Schizophrenia, Alcohol use disorders, Drug use disorders, Eating disorders, Diabetes mellitus, Chronic kidney diseases, Urinary diseases and male infertility, Gynaecological diseases, Hemoglobinopathies and Haemolytic anaemias, Endocrine, metabolic, blood, and immune disorders, Rheumatoid arthritis, Other musculoskeletal disorders, Congenital anomalies, Skin and subcutaneous diseases, Road injuries, Other transport injury, Falls, Drowning, Fire, heat and hot substances, Poisonings, Exposure to mechanical forces, Adverse effects of medical treatment, Animal contact, Foreign body, Self-harm, Interpersonal violence, Exposure to forces of nature |
| p025 | Proportionally | Sequelae of inflammatory diseases of central nervous system | G09-G090 | Meningitis, Encephalitis |
| p014 | Proportionally | Sequelae of other and unspecified infectious and parasitic diseases | A149 A29 A45-A459 A47 B93-B94 B948-B949 B956-B999 | Tuberculosis, HIV/AIDS, Diarrheal diseases, Intestinal infectious diseases, Meningitis, Encephalitis, Varicella and herpes zoster, Malaria, Other neglected tropical diseases, Sexually transmitted diseases excluding HIV, Hepatitis, Other infectious diseases, Cirrhosis, Skin and subcutaneous diseases |
| interm-151 | Proportionally | Shock, unspecified | R579 | Collective violence and legal intervention, Exposure to forces of nature, Maternal abortive outcome, Maternal haemorrhage, Other maternal disorders, Obstructed labour, Ischemic heart disease, Hypertensive heart disease, Chronic kidney diseases, Aortic aneurysm, Road injuries, Falls, Self-harm, Interpersonal violence, Exposure to mechanical forces |
| interm-60 | Fixed | Streptococcal, severe and other sepsis. Gas gangrene and gangrene not elsewhere classified.Toxic shock syndrome. Staphylococcal infection, unspecified site | A40-A419 A480 A483 A490 D65-D659 I96-I969 R02-R029 R652 | HIV/AIDS, Diarrheal diseases, Intestinal infectious diseases, Lower respiratory infections, Otitis media, Meningitis, Encephalitis, Measles, Varicella and herpes zoster, Maternal sepsis and other pregnancy related infection, Maternal abortive outcome, Sepsis and other infectious disorders of the newborn baby, Sexually transmitted diseases excluding HIV, Other infectious diseases, Endocarditis, Other cardiovascular and circulatory diseases, Appendicitis, Gall bladder and bile duct disease, Other digestive diseases, Urinary diseases and male infertility, Gynaecological diseases, Endocrine, metabolic, blood, and immune disorders, Other musculoskeletal disorders, Skin and subcutaneous diseases, Sense organ diseases, Road injuries, Other transport injury, Falls, Drowning, Exposure to mechanical forces, Adverse effects of medical treatment, Foreign body, Unintentional injuries not classified elsewhere, Self-harm, Interpersonal violence |
| interm-119 | Fixed | Toxic encephalopathy | G92-G929 | Diabetes mellitus, Chronic kidney disease, Cirrhosis, Pancreatitis |
| p047 | Proportionally | Unspecified transport accident | V99-V990 Y85-Y859 | Road injuries, Other transport injury, Drowning |
| interm-212 | Proportionally | Vesicointestinal, involving female genital tract or not elsewhere specifed fistulae | N321-N322 N82-N829 | Obstructed labour, Other maternal disorders, Sexually transmitted diseases excluding HIV, Cervical cancer, Uterine cancer, Colon and rectum cancer, Bladder cancer, Other malignant cancers, Other digestive diseases, Urinary diseases and male infertility, Gynaecological diseases, Endocrine, metabolic, blood, and immune disorders, Congenital anomalies, Skin and subcutaneous diseases, Adverse effects of medical treatment, Unintentional injuries not classified elsewhere |
| p008 | Proportionally | Viral infection of unspecified site | B34-B349 G933 | HIV/AIDS, Meningitis, Encephalitis, Varicella and herpes zoster, Hepatitis, Other infectious diseases, Cirrhosis |
| interm-56 | Fixed | Volume depletion. Hypo-osmolality and hyponatremia | E86-E869 E871 | Diarrheal diseases, Yellow fever, Protein-energy malnutrition, Eating disorders, Falls, Drowning, Fire, heat and hot substances, Poisonings, Exposure to mechanical forces, Adverse effects of medical treatment, Animal contact, Foreign body, Unintentional injuries not classified elsewhere, Self-harm, Exposure to forces of nature |
